# Characterization of gene expression patterns in response to an orthotospovirus infection between two diploid peanut species and their hybrid

**DOI:** 10.3389/fpls.2023.1270531

**Published:** 2023-11-13

**Authors:** Yi-Ju Chen, Michael A. Catto, Sudeep Pandey, Soraya Leal-Bertioli, Mark Abney, Brendan G. Hunt, Sudeep Bag, Albert Culbreath, Rajagopalbabu Srinivasan

**Affiliations:** ^1^ Entomology Department, University of Georgia, Griffin, GA, United States; ^2^ Plant Pathology Department, University of Georgia, Athens, GA, United States; ^3^ Institute of Plant Breeding, Genetics and Genomics, University of Georgia, Athens, GA, United States; ^4^ Entomology Department, University of Georgia, Tifton, GA, United States; ^5^ Plant Pathology Department, University of Georgia, Tifton, GA, United States

**Keywords:** *Arachis*, tomato spotted wilt orthotospovirus, transcriptomics, differential expression, gene ontology

## Abstract

Tomato spotted wilt orthotospovirus (TSWV) transmitted by thrips causes significant yield loss in peanut (*Arachis hypogaea* L.) production. Use of peanut cultivars with moderate field resistance has been critical for TSWV management. However, current TSWV resistance is often not adequate, and the availability of sources of tetraploid resistance to TSWV is very limited. Allotetraploids derived by crossing wild diploid species could help introgress alleles that confer TSWV resistance into cultivated peanut. Thrips-mediated TSWV screening identified two diploids and their allotetraploid possessing the AA, BB, and AABB genomes *Arachis stenosperma* V10309, *Arachis valida* GK30011, and [*A. stenosperma* × *A.* valida]^4x^ (ValSten1), respectively. These genotypes had reduced TSWV infection and accumulation in comparison with peanut of pure cultivated pedigree. Transcriptomes from TSWV-infected and non-infected samples from *A. stenosperma*, *A. valida*, and ValSten1 were assembled, and differentially expressed genes (DEGs) following TSWV infection were assessed. There were 3,196, 8,380, and 1,312 significant DEGs in *A. stenosperma*, *A. valida*, and ValSten1, respectively. A higher proportion of genes decreased in expression following TSWV infection for *A. stenosperma* and ValSten1, whereas a higher proportion of genes increased in expression following infection in *A. valida*. The number of DEGs previously annotated as defense-related in relation to abiotic and biotic stress was highest in *A. valida* followed by ValSten1 and *A. stenosperma*. Plant phytohormone and photosynthesis genes also were differentially expressed in greater numbers in *A. valida* followed by ValSten1 and *A. stenosperma*, with over half of those exhibiting decreases in expression.

## 1 Introduction

Tomato spotted wilt orthotospovirus (TSWV) is transmitted by thrips in a persistent propagative manner (Ullman, 1992). TSWV infection in peanut causes the spotted wilt disease (SWD). SWD has been the major concern in peanut production in the southeastern United States for the past three decades (Culbreath and Srinivasan, 2011; Srinivasan et al., 2017). Successful breeding efforts have led to the release of numerous peanut cultivars with moderate field resistance to TSWV (Culbreath and Srinivasan, 2011; Boukar et al., 2016). Peanut cultivars with moderate field resistance combined with other cultural practices have been instrumental in managing the SWD (Culbreath and Srinivasan, 2011; Srinivasan et al., 2017).

Field resistant peanut cultivars are not immune to the virus. They can be systemically infected with the virus and display TSWV characteristic symptoms upon infection (Srinivasan et al., 2017). The mechanism of field resistance to TSWV seems to be different in peanut than in other crops such as tomato and pepper, wherein resistance is governed by single dominant genes such as *Sw5, SlCHS3*, and *Tsw* (Stevens et al., 1991; Moury et al., 1997; Hoffmann et al., 2001; Lv et al., 2022; Lahre et al., 2023; Rodríguez-Negrete et al., 2023). In contrast, in peanut, five quantitative trait loci (QTLs) on chromosome A01 and one QTL on chromosome A09 have been found to be associated with TSWV resistance (Tseng et al., 2016; Zhao et al., 2018; Agarwal et al., 2019). The QTLs on A01 alone were responsible for 36% phenotypic variation associated with TSWV resistance, and A09 QTL contribution to TSWV resistance also was significant but not estimated (Tseng et al., 2016; Agarwal et al., 2019). Unlike tomato and pepper wherein the selection pressure induced by TSWV has led to resistance-breaking variants, no such resistance-breaking variants have been documented in peanut thus far (Sundaraj et al., 2014; Lai et al., 2021a). Therefore, it is likely that TSWV resistance in peanut is governed by multiple genes. Nevertheless, TSWV incidence in moderately field resistant cultivars is not robust and often dependent upon external factors such as vector and virus pressure. Peanut cultivars developed thus far with TSWV resistance are mostly from one peanut accession PI 203396 (Clevenger et al., 2018). The sources of TSWV resistance are extremely narrow, and reiterates the critical need to breed for robust TSWV resistance from other durable sources.

The *Arachis* genus is native to South America and contains 83 described species (Valls and Simpson, 2005; Valls et al., 2013; Santana and Valls, 2015; Valls and Simpson, 2017; Seijo et al., 2021). Many diploid accessions of *A. cardenasii* (Krapov. and W.C. Greg.), *A. correntina* ((Burkart) Krapov. and W.C. Greg.), *A. diogoi* (Hoehne), *A. villosa* (Bentham), and *A. stenosperma* (Krapov and W.C. Greg.) have exhibited resistance to TSWV (Lyerly et al., 2002). For instance, *A. diogoi* (GKP 10602) was identified as resistant to TSWV among 46 wild *Arachis* accessions (Milla et al., 2005; Lai, 2015; Stalker, 2017). Several QTLs linked to TSWV resistance have been mapped in wild diploid genotypes. Five markers for TSWV resistance were found from two AA genome wild species, *A. kuhlmannii* (Krapov. and W.C. Greg.) (VRGeSv 7639) and *A. diogoi* (GKP 10602) (Moretzsohn et al., 2013). In addition to TSWV, wild species also have been documented to confer resistance to its vector –thrips. Twelve diploid species were considered as potential sources for resistance to the thrips *Frankliniella fusca* (Hinds) (Stalker and Campbell, 1983; Lyerly et al., 2002), and antibiosis-based resistance to thrips was also found in *A. diogoi* and its hybrid (*A. hypogaea* × *A. diogoi*) (Lai, 2015; Srinivasan et al., 2018).

The cultivated allotetraploid peanut *Arachis hypogaea* (L.) (4n=40 chromosomes; AABB-type genome) was generated from the natural hybridization of two wild diploid species: *A. duranensis* (Krapov. and W.C. Greg.) (2n=20 chromosomes; AA-type genome) and *A. ipaensis* (Krapov. and W.C. Greg.) (2n=20 chromosomes; BB-type genome) (Husted, 1930). Additionally, genetic deletions and exchanges within and between the subgenomes of the progenitors have been found to be advantageous in domestication (Bertioli et al., 2016). Cultivated peanut is a self-pollinating crop with very low genetic variability (Moretzsohn et al., 2013). Consequently, resistance to TSWV and other pathogens is limited. On the contrary, several diploid wild species possess more resistance to TSWV and many other pathogens than cultivated peanut. However, transferring TSWV resistance across ploidy levels has been limiting due to hybrid incompatibility. Recent advancements have overcome such issues and have led to the development of allotetraploids from diploids via artificial hybridization (Simpson, 1991; Leal-Bertioli et al., 2015; Stalker, 2017). Such allotetraploids are increasingly being utilized in peanut breeding (Stalker, 2017; Chu et al., 2021).

In induced tetraploid genotypes, TSWV resistance conferring QTLs were located on chromosomes A03 and B08 in ValSten1, B05 and B10 in IpaCor, and A02, A05, and A06 in IpaCor (Levinson, 2021). More wild species related materials have been registered as TSWV resistant genotypes, such as ValSten1-GA-NC, IpaCor2-GA-NC, and IpaDur3-GA-NC (Chu et al., 2021). Next-generation sequencing (NGS) and transcriptome analysis have provided insights on virus-host interactions in TSWV susceptible and resistant peanut cultivars (Catto et al., 2021). Defense responses in general were overexpressed following TSWV infection, and more so in the case of TSWV-resistant cultivar than in the susceptible cultivar (Catto et al., 2021). The goal of this study was to develop transcriptomes and examine differential gene expression following TSWV inoculation in wild peanut. Candidate genotypes were selected based on phenotypic responses caused by thrips feeding and virus infection, whereby *A. stenosperma* and *A. valida*, and the resulting allotetraploid [*A. stenosperma* × *A. valida*]^4x^ (ValSten1) showed the lowest TSWV infection indices among the investigated genotypes in an associated study (Chen et al., 2023). Furthermore, the TSWV-induced gene expression changes in the selected wild species and their hybrid were compared with the expression changes of orthologs in the cultivated peanut genotypes.

## 2 Materials and methods

### 2.1 Maintenance of *Arachis* species plants

**Figure 1:**
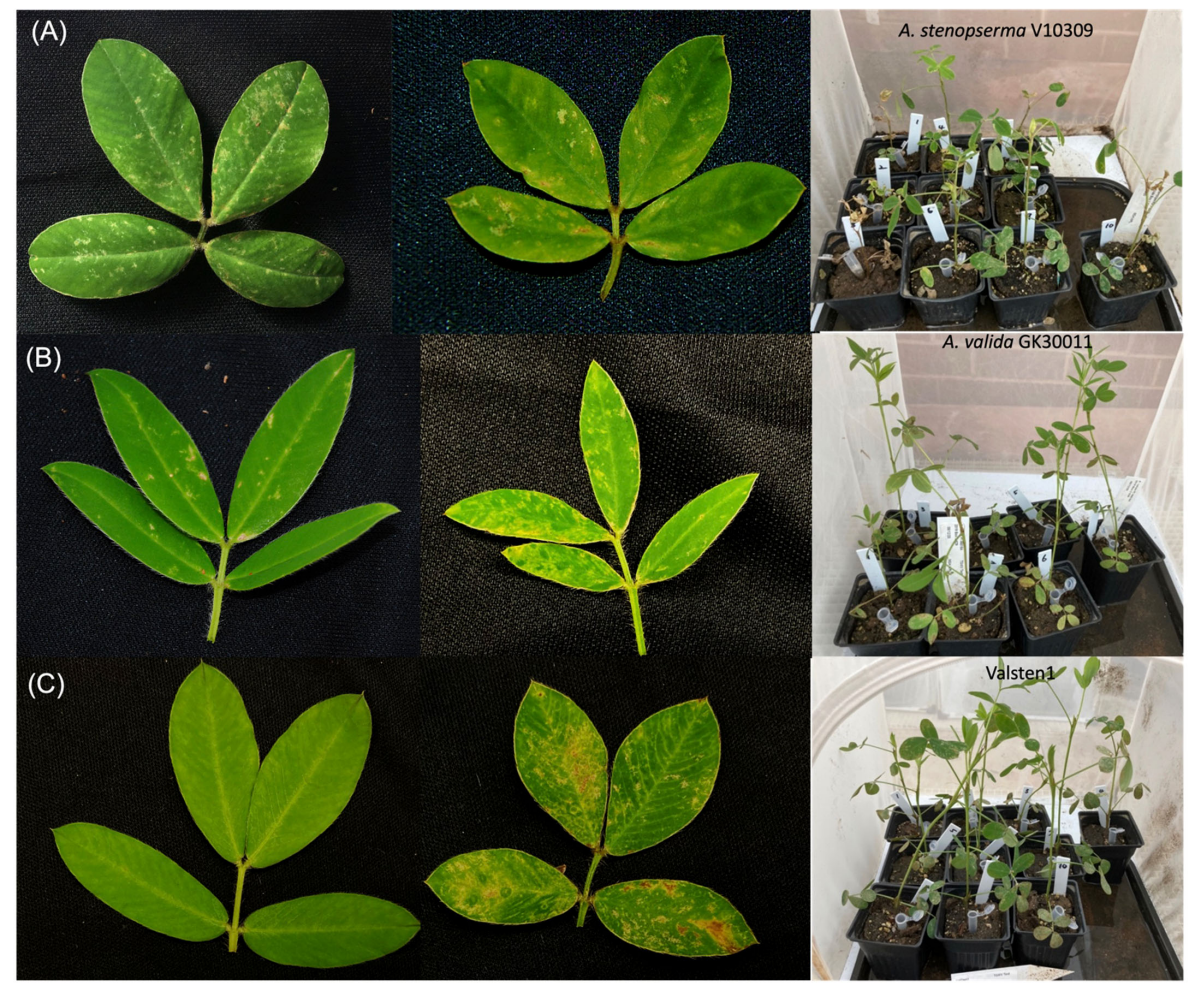
TSWV- induced symptoms on diploid *Arachis* species and their hybrid: **(A)** *A. stenosperma* V10309 **(B)** *A. valida* GK30011, and **(C)** the allotetraploid hybrid ValSten1 Left photograph represents a non-infected leaf, middle photograph represents a TSWV- infected leaf, and right photograph represents the whole plant after two weeks of thrips- mediated inoculation including infected and non-infected plants.

Two diploid species and their allotetraploid hybrid, namely *A. stenosperma* V10309 (PI666100) (**Figure 1A**), *A. valida* GK30011 (PI468154) (**Figure 1B**), and [*A. valida* GK30011 × *A. stenosperma* V10309 (PI695393)]^4x^ (**Figure 1C**) were used in this study (**Additional File 1:** **Figure S1**) (Chu et al., 2021; Gao et al., 2021; Chen et al., 2023). *A. valida* is a diploid species with the BB genome; *A. stenosperma* is a diploid species with the AA genome; and induced allotetraploid ValSten1 has AABB genome. Seeds of these genotypes were treated with two to three ml of a 0.5% solution of Florel^®^ Growth Regulator (Monterey Lawn and Garden, Fresno, California, USA) and incubated in a petri dish at 28°C for 18-24h to break seed dormancy. Seeds were sown in individual 4” pots with commercial potting mix Promix (Premier Horticulture Inc, Quakertown, PA, USA). The plants were kept in thrips-proof cages (47.5 cm^3^) (Megaview Science, Taichung, Taiwan) at 25-30°C, 80-90% RH, and a photoperiod of L14: D10 in the greenhouse. Seeds of the allotetraploid cultivar Georgia Green were pre-geminated in moistened paper towel and incubated in a growth chamber kept at 28°C for two to three days and used for thrips maintenance. One-to-two-week-old seedlings with one-to-two nodes and up to 16 leaflets of each genotype were used for TSWV transmission.

### 2.2 Development of *Arachis* hybrid ValSten1

The hybrid ValSten1 plants were developed based on the protocol described in Gao et al. (2021). Briefly, in the greenhouse, *A. valida* plants were emasculated and pollinated with fresh pollen of *A. stenosperma*. Hybrid plants were identified by a series of pollen traits and tests as described in Gao et al. (2021). Once the hybrid plants were identified, whole genome duplication using small 20-cm lateral branch sections and colchicine was undertaken. Cuttings and resulting plants were then maintained in the greenhouse as stated in Gao et al. (2021). Pods harvested from these plants were assessed by cytological and phenotypic analysis. Three morphological variations viz., flower width, branch angle, and pod wieight variations further confirmed the induced allotetraploid status of ValSten1 plants.

### 2.3 Thrips maintenance

Non-viruliferous thrips and viruliferous *Frankliniella fusca* thrips were maintained in separate growth chambers. Non-viruliferous thrips were maintained on leaflets of non-infected plants (cv. Georgia Green) within Petri dishes stuffed with a wet cotton round. Colonies were maintained by successive releases of ten adult female thrips, allowed to oviposit for 48h on a peanut leaflet dusted with a trace of pine pollen, and placed in growth chambers at 28-30°C and a photoperiod of L14: D10. Fresh leaflets and water were added to the Petri plates three times a week until emergence of the F_1_ generation. TSWV viruliferous thrips colony was maintained similarly on TSWV-infected leaflets collected from the field in a separate growth chamber as described previously (Shrestha et al., 2013). During the off-season, viruliferous thrips were maintained on TSWV-infected leaflets generated by mechanical inoculation in the greenhouse (Marasigan et al., 2015; Shrestha et al., 2015).

TSWV viruliferous and non-viruliferous nature of thrips colonies was periodically tested by RT-qPCR using N-gene-specific primers as previously described with appropriate controls (Rotenberg et al., 2009; Shrestha et al., 2012; Shrestha et al., 2017). At each instance, a subset (~ten each) of viruliferous and non-viruliferous thrips were evaluated for TSWV infection status. All the viruliferous thrips evaluated tested positive and all the non-viruliferous thrips tested negative for TSWV. These indicated that the thrips colonies were true to their infection status or lack thereof.

### 2.4 Thrips-mediated inoculation of diploids and their hybrid

*F. fusca*-mediated inoculation was conducted as per the established protocol previously (Shrestha et al., 2015). The experiment included two treatments: mock inoculation via non-viruliferous *F. fusca* thrips (non-infected) and TSWV inoculation via viruliferous thrips (TSWV-infected). Inoculated plants were maintained in thrips-proof cages (47.5 cm^3^) in the growth chamber at 27°C and ~80% humidity (Conviron, Pembina, ND, USA). After two weeks, the first fully expanded leaf of inoculated peanuts (ca 0.03 g) was tested by RT-qPCR following methods described previously (Shrestha et al., 2015; Chen et al., 2023) to assess TSWV-infection status.

### 2.5 Sample preparation, total RNA extraction, and quality control

Samples from plants two-to-three weeks post-inoculation were used. Five replications for each genotype were used. Leaflets were collected from the first fully expanded leaf below the terminal of each plant for RNA extraction. Total RNA was extracted by RNeasy plant mini kit following the manufacturer’s protocol (Qiagen, Valencia, CA, UGA). For each replicate, a leaflet sample was obtained from an individual plant. Thus, a total of 30 RNA samples were prepared for sequencing (three genotypes × two infection status × five replicates) and were stored at -80°C before shipping. Prior to library preparation, each sample’s integrity (RNA integrity number, RIN) was measured by using Aglient 2100 Bioanalyzer (Agilent Technologies, Santa Clara, CA, USA) for RNA quality control (QC). Two samples failed the QC test; therefore 28 samples were used for library preparation and sequencing.

### 2.6 Library preparation and sequencing

The complementary DNA (cDNA) synthesis, cDNA libraries (messenger RNA library), and sequencing were undertaken by Novogene Corporation Inc. (Sacramento, CA, USA), as described in Catto et al. (2021). Illumina sequencing libraries were constructed using TruSeq RNA sample preparation kits. Briefly, mRNA was selected, fragmented, and first-strand cDNA was synthesized using random primers and reverse transcriptase. Subsequently, Polymerase I and RNase H were used to make the second-strand cDNA. An Illumina TruSeqLT adapter was ligated to the DNA fragments, and PCR amplification was performed for a minimal number of cycles with standard Illumina primers to produce the final cDNA libraries. Twenty-eight libraries were constructed and sequences using two lanes in the Illumina NovaSeq 6000 platform (pair-end 150 cycle sequencing setting, > 6GB raw data per sample).

### 2.7 Raw read processing for transcript abundance

In advance of the *A. valida*, *A. stenosperma*, and ValSten1 transcriptome assemblies (**Additional File 1:** **Figure S2**), FastQC v0.11.9 and multiQC v1.11 were used to check the quality of raw reads before and after trimming (Andrews, 2010; Ewels et al., 2016). Trimmomatic v0.39 software was used with the default setting to remove adapters (Bolger et al., 2014). Also, Sortmerna v4.3.3 software was used with the SILVA database to remove rRNA contamination (Kopylova et al., 2012; Yilmaz et al., 2014; Glöckner et al., 2017). The rRNA decontaminated trimmed reads were converted from interleaved to paired files using BBMap v38.93 software for configuring files and for transcriptome assembly (Bushnell, 2014).

### 2.8 Transcriptome assembly pipeline and quality control

The rRNA decontaminated and trimmed reads from *A. valida*, *A. stenosperma*, and ValSten1 were used to generate respective *de novo* assemblies using Trinity v2.10.0 software with the default parameters (Grabherr et al., 2011). The sra2genes v4 software was used to clean up the assemblies using prior evidence from closely related species to address the possibility of over assembly of the transcriptome. Sra2genes is a complete pipeline to reconstruct genes from RNA data sources, and it includes several tools such as Cluster Database as High Identity of Tolerance (CD-HIT) v4.8.1, Exonerate v2.4.0, Blast+ 2.10.1, and A Genomic Mapping and Alignment Program for mRNA and expressed sequence tag (EST) Sequences – Genomic Short-read Nucleotide Alignment Program (GMAP-GSNAP) (Slater and Birney, 2005; Fu et al., 2012; Wu et al., 2016). CD-HIT v4.8.1 was used for the removal of potentially chimeric or misassembled transcripts from the input reads. Exonerate v2.4.0 was involved in the removal of all duplicated sequences. Blast+ 2.10.1 was used to separate the transcripts as various isoforms. GMAP-GSNAP was used to align the reads to the assemblies. Benchmarking Universal Single Copy Orthologs (BUSCO) v4.0.6 was used to determine assembly completeness before and after cleaning of the *de novo* assemblies against the Fabales odb10 lineage (n=5,366) (Simão et al., 2015; Seppey et al., 2019; Manni et al., 2021).

### 2.9 Mapping of reads and differential expression

Trimmed reads were mapped to the respective *de novo* assemblies (see Data Availability for NCBI assessions) using Bowtie2 v2.4.1 with default mapping parameters (Langmead et al., 2009; Langmead and Salzberg, 2012; Langmead et al., 2019). Gene count estimates were derived from the mapped reads using RNA-Seq by Expectation Maximization (RSEM) v1.3.3 for *A. stenosperma* (**Additional File 2:** **Table S1**), *A. valida* (**Additional File 2:** **Table S2**), and ValSten1 (**Additional File 2:** **Table S3**) (Li and Dewey, 2011). Custom R script was used to determine the fragments per kilobase million (FPKM) across all samples on R v4.1.0 using the following R libraries: dplyr, tidyverse, and stringr (**Additional File 1:** **Figures S3–S5**) (R Core Team, 2021). DESeq2 was used to measure differentially expressed genes by comparing the gene counts from non-infected samples with virus-infected samples, where genes that had a |log_2_ fold change (LFC)| ≥ 4 and a false discovery rate (FDR) < 0.05 were classified as being significantly differentially expressed (Love et al., 2014).

### 2.10 Functional annotation

The *de novo* assemblies for *A. stenosperma* (**Additional File 3:** **Table S4**), *A. valida* (**Additional File 3:** **Table S5**), and ValSten1 (**Additional File 3:** **Table S6**) were compared against an *Arachis* filtered subset of the NCBI database for non-redundant proteins (NR) and RefSeq genes using OmicsBox (Götz et al., 2008; Camacho et al., 2009). The OmicsBox tool also performed Blast2GO and Gene Ontology (GO) mapping to assign functional annotations to genes within each assembly (Conesa et al., 2005; Götz et al., 2008; Mi et al., 2019). Additional annotations were performed using InterProScan and the Kyoto Encyclopaedia of Genes and Genomes (KEGG) (Kanehisa and Goto, 2000; Jones et al., 2014; Kanehisa et al., 2016). The GO terms were processed with topGO (https://www.bioconductor.org/packages/release/bioc/html/topGO.html) and visualized using rrvgo (https://bioconductor.org/packages/release/bioc/html/rrvgo.html) and the reduced + visualize Gene Ontology (REVIGO) web tool (Supek et al., 2011). GO terms down to level 3 were analysed.

### 2.11 Clustering of differentially expressed genes into orthogroups

DEGs from two wild peanut species: *A. stenosperma* and *A. valida*, their respective hybrid ValSten1, and previously published DEGs from two domestic peanut cultivars: *A. hypogaea* (SunOleic 97R) and *A. hypogaea* (Tifguard) (Catto et al., 2021) were used to determine DEG clusters using the online tool OrthoVenn2 (Xu et al., 2019). The parameters for DEG ortholog clustering in OrthoVenn2 were run with the cut-off value of 1e^-5^. Overlapping regions were tested for significance using GeneOverlap (https://bioconductor.org/packages/release/bioc/vignettes/GeneOverlap/inst/doc/GeneOverlap.pdf).

### 2.12 Validation of RNA sequence using RT-qPCR

Quantitative reverse transcription-polymerase chain reaction (RT-qPCR) was utilized to validate *Arachis* species transcripts following TSWV infection. Three sequences from each genotype with a |LFC| ≥4 and a false discovery rate (FDR) < 0.05 were randomly selected. The sequences were extracted with the tool seqtk. RT-qPCR was performed on plant samples obtained from four biological repeats from the remaining samples. Primers for targeted DEGs were designed by NCBI primer design (https://www.ncbi.nlm.nih.gov/tools/primer-blast/). Primer sequences are listed in **Additional File 1:** **Table S7**.

The cDNA was synthesized by a Go-Script reverse transcription system (Promega Corporation, Madison, WI) following the manufacturer’s protocol and then diluted 20-fold for quantitative polymerase chain reaction (qPCR). The reaction mix for qPCR included 2x GoTaq qPCR Master Mix, 1 μl of sequence-specific primers (final concentration of 250 mM), 2 μl cDNA of sample, and nuclease-free water for a final reaction volume of 20 μl. The reaction was run at 95°C for 2 min, followed by 40 cycles at 95°C for 15s, 58°C for 20s, and 72°C for 30s. The reaction was extended with a melting curve in a QuantStudio 3 System (applied biosystems by Thermo Fisher Scientific, Waltham, MA) to rule out non-specific binding. Two technical replicates for targeted transcripts and the reference gene (alcohol dehydrogenase class III) (Lai et al., 2021b), and water control were included in each RT-qPCR run. The log_2_fold change of each target transcript in infected plants against mock-inoculated plants was calculated after normalization to the reference gene. The log_2_ transformed (ratio of infected samples/ratio of non-infected samples) expression of target genes (transcripts) were correlated with Pearson’s correlation using the function “cor” in software R.

## 3 Results

### 3.1 Transcriptome assembly and sequencing statisitics

Total raw reads obtained from infected plants and non-infected plants of *A. valida* GK30011 (PI468154), *A. stenosperma* V10309 (PI666100), and ValSten1 were assembled *de novo* using Trinity platform. Total raw reads generated from the three genotypes were 222, 234, and 253 million pair reads, respectively, which after trimming amounted to 218, 231, and 250 million pair reads, respectively. The percentage of reads mapped to the *de novo* assembled transcriptome for *A. stenosperma*, *A. valida*, and ValSten1 genotypes were 86%, 87%, and 80%, respectively (**Additional File 1:** **Table S8**). These reads were assembled into 141,144 (*A. valida*), 106,374 (*A. stenosperma*), and 137,039 (ValSten1) contigs. The assembly of *A. stenosperma* contained 4,571 (85%) complete BUSCOs, which included 2,571 (48%) single-copy and 2,000 (37%) duplicated orthologs. Similarly, *A. valida* contained 4,724 (88%) complete BUSCOs, which included 2,545 (47%) single-copy and 2,179 (41%) duplicated orthologs. For ValSten1, there were 4,670 (87%) complete BUSCOs, which included 2,209 (41%) single-copy and 2,461 (46%) duplicated orthologs. One infected sample of *A. stenosperma* showed low RIN (RNA integrity number) and one non-infected sample of *A. valida* that showed uneven baseline at QC were not processed from the initial 30 libraries.

### 3.2 Quantitation of differential expression analysis profile

**Figure 2:**
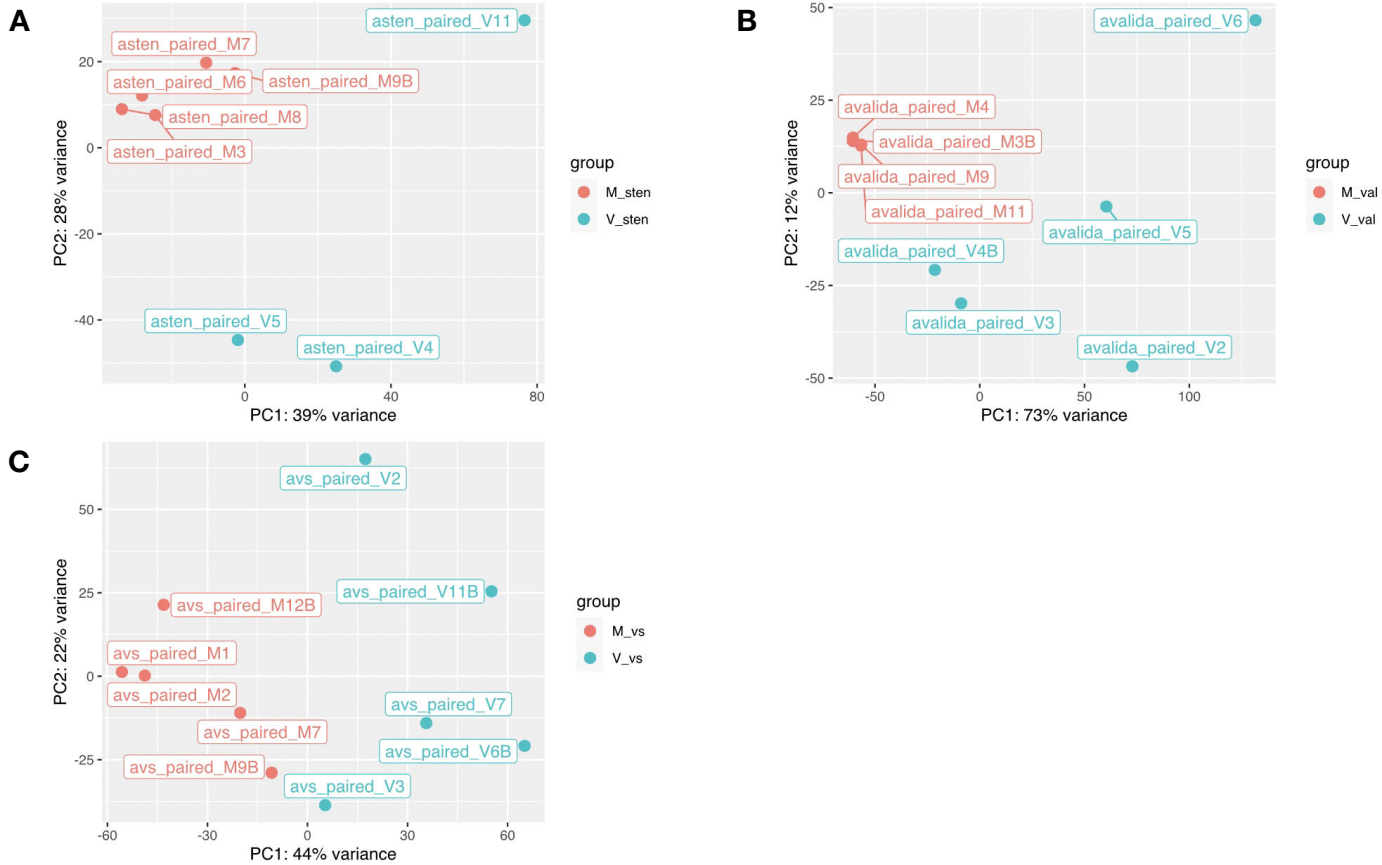
Principal component analysis based on the gene expression levels in two diploid Arachis species and their hybrid. **(A)** *A. stenosperma* V10309, **(B)** *A. valida* GK30011, and **(C)** ValSten1 clustered together according to being either non-inoculated (M, in red color) or TSWV-infected (V, in blue color). .

**Figure 3:**
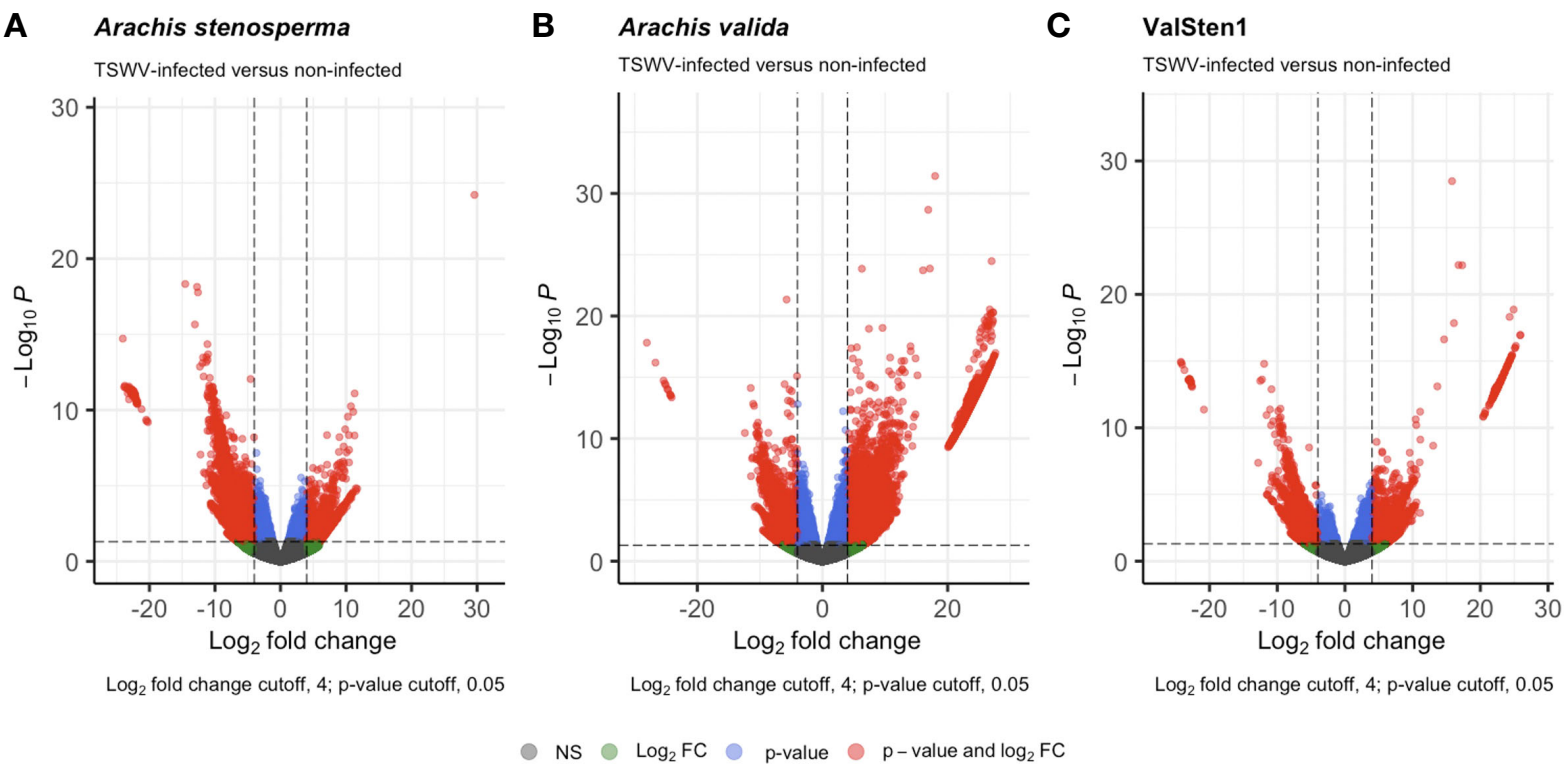
Volcano plots detailing the differential expression profiles of TSWV-infected versus non-infected samples of two diploid Arachis species and their hybrid. Genes with a |LFC| >4 and a false discovery rate (FDR) < 0.05 are highlighted in red were considered to be differentially expressed: **(A)** 3,196 DEGs from *A. stenosprema* V10309 (PI666100), **(B)** 8,380 DEGs from *A. valida* GK30011 (P1468154), and **(C)** 1,312 DEGs from ValSten1 (P1695393).

The reads obtained from infected and non-infected samples from the three genotypes were normalized and clustered using FPKM and principal component analysis (PCA) for comparison. The PCA clustered TSWV infected samples of the three genotypes separately from the non-infected ones (**Figure 2**). However, one sample (asten_paired_V3B) in *A. stenosperma* was removed due to the unexpected clustering in PCA, although it did not have a reduced FPKM value (**Additional File 1:** **Figure S6**). Additional checks on infection status were performed by mapping reads, using RSEM and Bowtie2, from *A. stenosperma* and *A. valida* to the ValSten1 *de novo* assembly and clustering the samples via PCA (**Additional File 1:** **Figure S7**). Differentially expressed genes (DEGs) observed for *A. stenosperma*, *A. valida*, and ValSten1 in response to TSWV were 3,196 (596 overexpressed and 2,627 underexpressed; **Figure 3A**), 8,380 (6,332 overexpressed and 2,048 underexpressed; **Figure 3B**), and 1,312 (633 overexpressed and 679 underexpressed; **Figure 3C**), respectively. TSWV-infected samples of *A. valida* had more DEGs (8,380) compared with *A. stenosperma* (3,196) and ValSten1 (1,312). A higher percentage of DEGs for *A. stenosperma* were underexpressed, whereas more overexpressed genes were identified in *A. valida*. Similar numbers of underexpressed and overexpressed genes were found within ValSten1.

### 3.3 Functional annotation of genes

DEGs observed in the wild species in response to TSWV infection were functionally annotated. The *de novo* assemblies included 107,043 transcripts, 149,877 transcripts, and 138,389 transcripts (non-significant and significant genes) of *A. valida*, *A*. *stenosperma*, and ValSten1, respectively.

Gene ontology (GO) provided context for the functionality of genes and comprised three level 1 categories: biological process (BP), cellular component (CC), and molecular function (MF). GO terms within the BP category provided biological relevance by attributing biological objectives to gene products. Significantly enriched GO terms were determined by the Revigo tool (Supek et al., 2011) by comparing the GO terms distribution from DEGs to that of the entire transcriptome, also referred to as the background genes. DEG specific GO terms that were overrepresented were considered significantly enriched (p < 0.05) with respect to the background.

**Figure 4:**
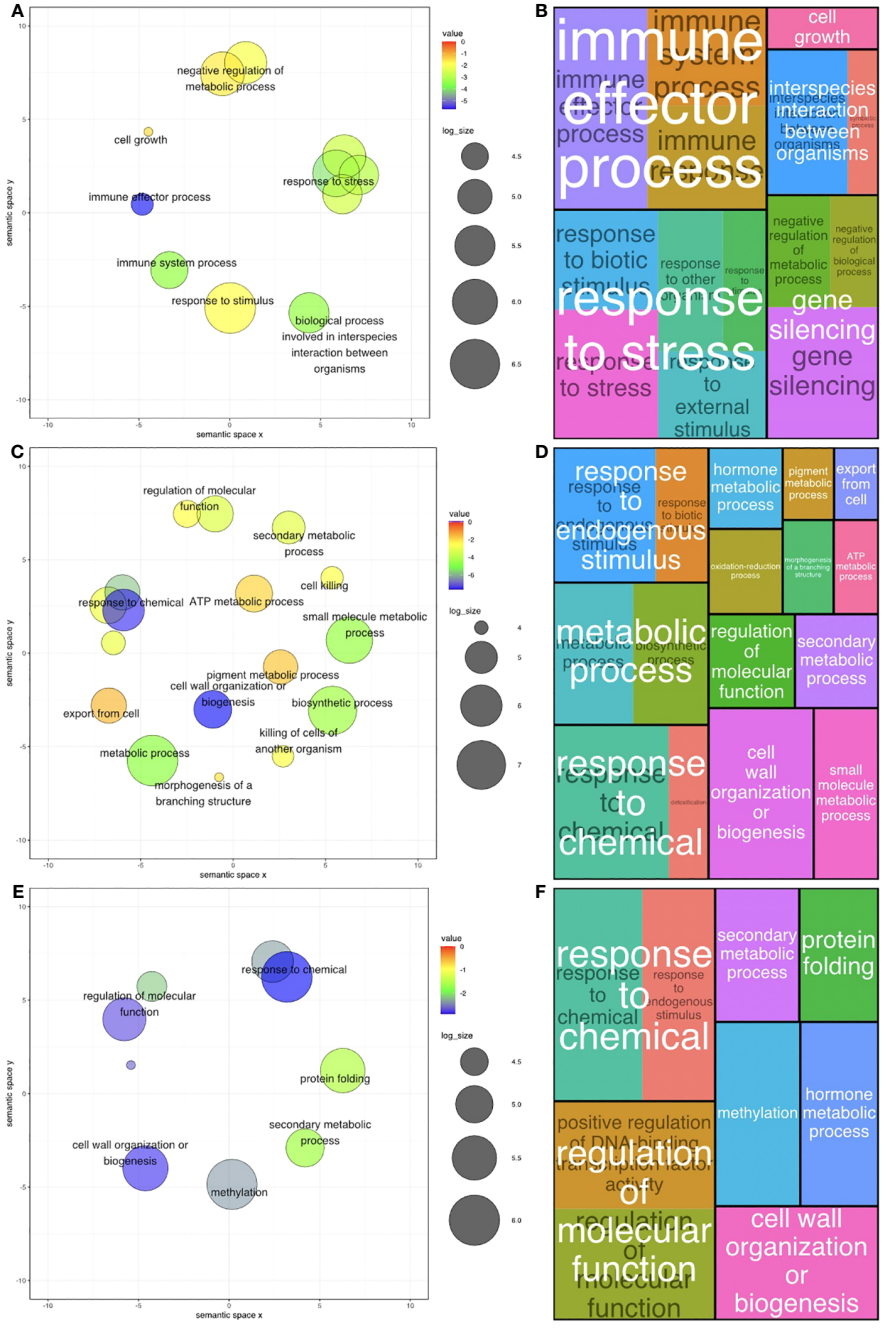
Gene Ontology (GO) level 2 & 3 terms ratios across two diploid *Arachis* species and their hybrid. **(A)** Ratio of all significant GO terms assigned to differentially expressed genes (DEGs) present in *A. stenosperma*. **(B)** Tree map of significant levels 2 & 3 GO terms of DEGs compared to the background in *A. stenosperma*. **(C)** Ratio of all significant GO terms assigned to differentially expressed genes (DEGs) present in *A. valida* **(D)** Tree map of significant levels 2 & 3 GO terms of DEGs compared to the background in *A. valida*. **(E)** Ratio of all significant GO terms assigned to differentially expressed genes (DEGs) present in ValSten1 **(F)** Tree map of significant levels 2 & 3 GO terms of DEGs compared to the background in ValSten1.

In *A. stenosperma*, 127 BP GO terms were significantly enriched among DEGs across all GO term levels (**Additional File 1:** **Figures S8A, B**; **Additional File 4:** **Tables S9, S10**), with 14 terms being classified as levels 2 & 3 (**Figures 4A, B**). In *A. valida*, 256 BP GO terms were significantly enriched among DEGs across all GO term levels (**Additional File 1:** **Figures S8C, D**; **Additional File 4:** **Tables S11, S12**), with 19 terms being classified as levels 2 & 3 (**Figures 4C, D**). In ValSten1, 135 BP GO terms were significantly enriched among DEGs across all GO term levels (**Additional File 1:** **Figures S8E, F**; **Additional File 4:** **Tables S13**, **S14**), with 9 terms being classified as levels 2 & 3 (**Figures 4E, F**).

### 3.4 Comparison of DEGs between genotypes

**Figure 5:**
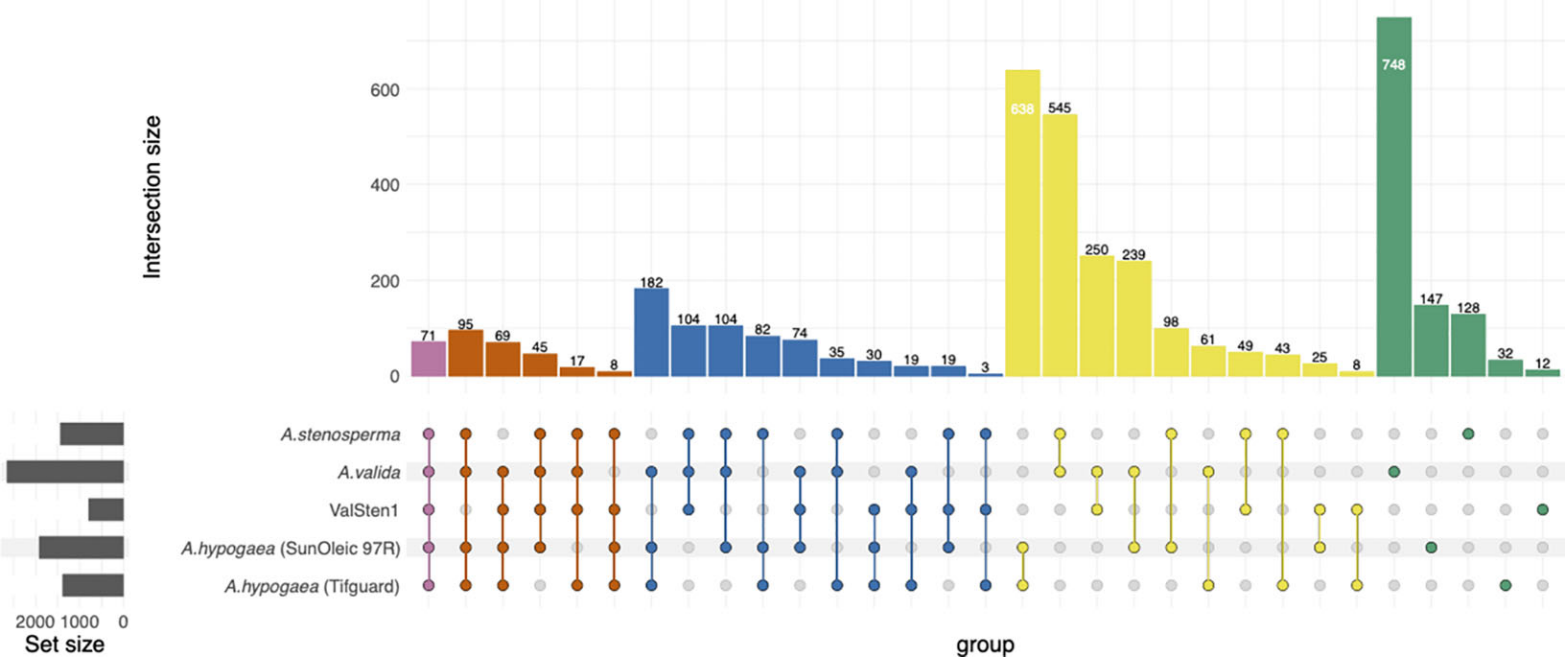
Distribution of shared peanut (*Arachis spp.*) gene families containing expressed genes in response to TSWV infection. Venn diagram represents the expressed common, unique, and core set of DEGs within gene families between *A. stenosperma, A. valida*, ValSten1 (*A. valida x A. stenosperma*), *A. hypogaea* (SunOleic 97R), and *A. hypogaea* (Tifguard).

To determine the transcriptional changes in each genotype related to TSWV infection, the number of orthologous clusters between *A. stenosperma*, *A. valida*, ValSten1, *A. hypogaea* (SunOleci 97R), and *A. hypogaea* (Tifguard) (Catto et al., 2021) were compared using the OrthoVenn2 web platform (**Figure 5**). Orthologous clustering analysis resulted in 3,965 clusters of DEGs that were commonly shared by at least two genotypes (**Additional File 5:** **Table S15**) and 15 single-copy DEG clusters from all five genotypes (**Additional File 5:** **Table S16**). In total, 71 DEG clusters were found to contain DEGs shared between all five genotypes, with cluster53, cluster179, and cluster185 relating to the putative disease resistance protein RGA3 (Song et al., 2003; Van Der Vossen et al., 2003) (UniProt ID: Q7XA40) and defense response (GO:0006952; **Additional File 5:** **Table S15**). There were 17 DEG clusters that comprised four of the genotypes, but not in the susceptible *A. hypogaea* (SunOleic 97R), with cluster674 relating to the TMV resistance protein N (UniProt ID: Q40392) (Whitham et al., 1994; Dinesh-Kumar and Baker, 2000; Dinesh-Kumar et al., 2000; Caplan et al., 2008) and signal transduction (GO:0007165; **Additional File 5:** **Table S15**). The highest overexpressed gene, with a LFC of 29.6, was found in *A. stenosperma* and was annotated as linoleate 9S-lipoxygenase (ArasteEVm001500t4). Manual assessment determined that such a large LFC was caused by lack of mapped reads (no detectable expression) in the mock inoculated/non-infected samples. This gene was found to be in cluster4, containing genes from all genotypes, and was functionally annotated as linoleate 9S-lipoxygenase (P38414) (Hilbers et al., 1994) and oxylipin biosynthetic process (GO:0031408; **Additional File 5:** **Table S15**).

With respect to *A. stenosperma*, *A. valida*, and ValSten1, orthologous DEG clustering analysis resulted in 1,507 DEG clusters that were commonly shared by at least two genotypes: *A. stenosperma* ⋂ *A. valida* (779), *A. stenosperma* ⋂ ValSten1 (79), *A. valida* ⋂ ValSten1 (412), or *A. stenosperma* ⋂ *A. valida* ⋂ ValSten1 (237) (**Additional File 1:** **Figure S9**). Additionally, 1,574 DEG clusters were found to be specific to *A. stenosperma* (269), *A. valida* (1,230), and ValSten1 (75) (**Additional File 1:** **Figure S9**). Sixty nine of the 237 orthologous clusters shared by the three wild peanut genotypes were reported as containing single-copy DEGs (**Additional File 5:** **Table S17**). All pairwise comparisons of DEG clusters from *A. stenosperma* ⋃ *A. valida* (2,404), *A. stenosperma* ⋃ ValSten1 (1,439), *A. valida* ⋃ ValSten1 (2,357) showed more overlap than expected by chance (Fisher’s exact test) p=1.1e^-50^, p=3.5e^-12^, and p=7.4e^-47^, respectively.

The phytovirus response DEGs from *A. stenosperma* (3,196), *A. valida* (8,380), and ValSten1 (1,312) were grouped into three major categories: defense, phytohormone, and photosynthesis related genes (**Table 1**). The categories were chosen based on the study with resistant and susceptible cultivated peanuts (Catto et al., 2021). Within the defense related DEGs, the percentage (No. of overexpressed DEGs out of total DEGs within category) in *A. stenosperma, A. valida*, and ValSten1 were 34% (25/73), 64% (490/763), and 55% (69/126), respectively (**Table 1**). A similar pattern was observed in the case of phytohormone related DEGs. Upregulation of phytohormone related DEGs of the eight examined categories was higher in *A. valida*. The percentages (No. of overexpressed DEGs out of total DEGs within category) in *A. stenosperma, A. valida*, and ValSten1 were 9% (1/11), 51% (100/198), and 36% (16/44), respectively (**Table 1**). Regarding photosynthesis related DEGs, the percentages (No. of overexpressed DEGs out of total DEGs within category) in *A. stenosperma, A. valida*, and ValSten1 were 10% (3/29), 46% (249/536), and 38% (28/73), respectively.

**Table 1:** Counts of defense-, phytohormone-, and photosynthesis-related significant differentially expressed genes with a |LFC| > 4 and a false discovery rate (FDR) < 0.05 cutoff in wild *Arachis* species in response to TSWV infection.

Gene description	*A. stenosperma* (Sten)		*A. valida* (Val)		ValSten1	
	Overexpressed	Underexpressed	Overexpressed	Underexpressed	Overexpressed	Underexpressed
Argonaute	0	0	1	6	0	0
MATH domain	0	0	1	1	0	0
Dicer	0	1	3	1	0	0
Heat shock protein	11	0	49	1	15	0
Lectin	1	2	47	12	12	2
Leucine zipper	1	0	10	4	3	1
Mitogen-activated protein kinase	0	1	13	4	0	0
MYB	1	2	23	13	2	2
P450	1	4	51	18	6	8
PAMP	0	0	1	0	0	0
Disease resistance (R) protein	1	12	11	49	4	13
WRKY transcription factor	0	1	22	3	2	0
LRR	1	4	24	25	1	2
Serine/threonine	7	11	129	89	14	23
Salicylic acid	0	0	3	1	0	0
Calmodulin	0	1	25	12	3	0
TMV resistance protein N	1	8	25	20	2	5
Stilbene synthase	0	0	33	0	0	0
Serine Carboxypeptidase	0	1	19	11	5	0
Alpha-Dioxygenase	0	0	0	3	0	1
(Total of genes related to defense)	(25)	(48)	(490)	(273)	(69)	(57)
Auxin	0	1	7	37	3	14
Gibberellin	0	0	5	8	1	3
Cytokinin	0	0	13	4	2	2
Abscisic acid	0	1	5	8	0	0
Ethylene	0	1	29	9	5	1
Brassinosteroid	0	0	0	0	1	0
Salicylic acid	0	0	0	0	1	0
ABC transporter	1	7	41	32	3	8
(Total of genes related to phytohormones)	(1)	(10)	(100)	(98)	(16)	(28)
Chloroplastic	3	23	243	256	26	44
Protochlorophyllide	0	0	1	3	0	0
Photosystem	0	1	0	26	0	1
NADP-dependent malic enzyme	0	2	5	2	2	0
(Total of genes related to phytosynthesis)	(3)	(26)	(249)	(287)	(28)	(45)

### 3.5 Validation of RNA-sequencing

Three DEGs from each genotype were randomly selected and their expression values were validated using RT-qPCR (**Additional File 1:** **Table S7**). A positive correlation was found between the expression from both RNASeq and RT-qPCR across all three genotypes (cor=0.87, t=4.6, df=7, *p*=0.002; **Additional File 1:** **Figure S10**).

## 4 Discussion

Peanut production could be severely impacted by orthotospoviruses such as TSWV (Culbreath et al., 2003; Culbreath and Srinivasan, 2011). Resistance against the pathogen and/or the vector is often the ideal management option. The cultivated peanut has a narrow genetic base due to relatively recent polyploidization and self-pollination (Pandey et al., 2012). Therefore, peanut genetics is prohibitive to crop improvement and/or enhancing pathogen resistance. While wild species can confer increased resistance against pathogens such as orthotospoviruses, introgressing that resistance into cultivated peanut is challenging mainly due to ploidy level differences (wild species are typically diploids) (Pandey et al., 2012; Bertioli et al., 2016). Several wild species have been recognized for innate resistance against orthotospoviruses, particularly TSWV (Moretzsohn et al., 2013; Lai, 2015; Stalker, 2017). Ability to induce allotetraploid hybrids from wild species with the same genetic makeup as the cultivated peanut, *A. hypogaea* (AABB genome), has allowed for transferring useful genes and increasing the genetic diversity of tetraploid peanut (Gao et al., 2021). As a part of continuing effort, numerous wild species and their hybrids were evaluated at the University of Georgia (Chen et al., 2023). The evaluations indicated that wild diploids such as *A. stenosperma* and *A. valida* and their allotetraploid hybrid, ValSten1, had reduced TSWV infection and accumulation than other diploids and the cultivated tetraploid evaluated following thrips-mediated inoculation (Chen et al., 2023). The severity of TSWV-induced symptoms also was reduced on *A. stenosperma* and *A. valida* and their allotetraploid hybrid than on the cultivated tetraploid (Chen et al., 2023).

To gain insights on interactions of *A. stenosperma* and *A. valida* and their allotetraploid hybrid with TSWV, gene expression patterns post thrips-mediated TSWV inoculation were examined in this study. Following thrips-mediated TSWV inoculation, based on *de novo* transcriptome assemblies, gene expression was substantially higher in *A. valida* than in *A. stenosperma* and ValSten1. Overall, in this study, expression of defense-related genes and genes associated with plant physiology such as phytohormones and photosynthesis were examined. Numerous genes pertaining to defense against biotic stress, including pathogens, were overexpressed in *A. valida* (BB genome) than in *A. stenosperma* (AA genome) following TSWV infection.

A greater proportion of contigs associated with pathogen defense such as heat shock proteins, lectins, and leucine zippers were overexpressed in *A. valida* followed and *A. stenosperma*. A heat shock protein was associated with virus infection in *Arabidopsis thaliana* (Roux and Bergelson, 2016). Lectins were known to upregulate plant defenses by facilitating recognition of phytoviruses (Fliegmann et al., 2004). Nucleotide binding-leucine rich repeats (NB-LRR) were known to provide defense against a range of pathogens including phytoviruses (Noman et al., 2017; Mishra et al., 2019; Zhang et al., 2023). A greater proportion of NB-LRR genes were overexpressed in *A. valida* than in *A. stenosperma* and in their hybrid in this study. Similarly, NB-LRR genes were overexpressed in a TSWV resistant tetraploid peanut cultivar than the susceptible tetraploid cultivar following TSWV infection (Catto et al., 2021). NB-LLR genes also were overexpressed in response to TSWV infection in TSWV-resistant tomato lines in another study (Lv et al., 2023). The overexpression of defense genes following thrips-mediated TSWV inoculation in this study provides mechanistic reasons for the observed response against TSWV in *A. valida*.

A suite of other defense genes such as calcium-modulated calmodulin, stilbene synthase, and serine carboxypeptidases also were overexpressed substantially in the case of *A. valida* followed by the hybrid, and *A. stenosperma*. These genes have been documented to mediate resistance against a wide array of pathogens including phytoviruses (Fraser et al., 2005; Yu et al., 2005; Takabatake et al., 2007; Hong et al., 2017; Catto et al., 2021). The differential gene expression pattern seems to be consistent, wherein defense genes’ upregulation in *A. valida* was almost always higher than in the hybrid and least in the other diploid, *A. stenosperma*. In addition, induced defense response related genes such as those associated with RNA interference and salicylic acid were overexpressed in a similar pattern in *A. valida* followed by the hybrid and *A. stenosperma*.

Besides the above-stated categories of genes, dominant genes that confer hypersensitive response were overexpressed in *A. valida* than in the other two genotypes. Hypersensitive response inducing genes such as nucleocapsid (N) gene from tobacco (*Nicotiana glutinosa* L.), which imparts resistance to several tobamoviruses including the tobacco mosaic virus (TMV), and disease resistance (R) proteins, were underexpressed in three resistant wild genotypes in this study. However, the R proteins were overexpressed in two cultivated genotypes in a previous study (Catto et al., 2021). In pepper, *Tsw* was the only identified R gene against TSWV (Wu et al., 2023), and Sw5 in tomato conferred hypersensitive response against TSWV (de Oliveira et al., 2018). Similarly, in tomato, the disease- resistant R gene *Mi* conferred resistance against nematodes and potato aphids (Rossi et al., 1998). However, HR can be uncoupled with resistance and may vary depending on species in some cases (Balint-Kurti, 2019). Perhaps this explains the absence of hypersensitive response in peanut following TSWV infection. Generally, R protein in plants recognizes the effectors in pathogens and are known to trigger a defense response. WRKY transcription factors also were involved in triggering immunity against a range of pathogens including viruses by recognizing pathogen associated molecular patterns (PAMPs) (Pandey and Somssich, 2009; Lee et al., 2023). WRKY was overexpressed in a tomato genotype with resistance to TSWV (Catoni et al., 2009; Lv et al., 2023). Similarly, WRKY contigs were substantially overexpressed in *A. valida* and slightly in the hybrid.

The results in the current study clearly illustrate that several classes of defense genes were overexpressed in *A. valida* (BB genome) and its hybrid ValSten1 (AABB genome). However, the obtained results were in contrast with previous studies, which showed wild species such as *A. stenosperma* and *A. cardenasii* with AA genomes harbored more defense genes’ containining QTLs than the wild species with the BB genomes (Bertioli et al., 2016; Pandey et al., 2017). The results from the current study indicate that the resistance to TSWV in wild peanut may have interspecific differences and need to be further examined in depth. Also, the current study was conducted at one time point, *i.e.*, three weeks post inoculation. Time-series profiling of DEGs will be beneficial for better understanding the changing pattern of gene expression in relation to TSWV infection. Further, not many studies thus far have evaluated gene expression in wild peanut species following TSWV infection, especially following thrips-mediated inoculation. Perhaps, some of these differences could explain the observed expression profiles of defense genes associated with the BB genome in *A. valida* as opposed to the AA genome in *A. stenosperma*. Despite this reoccurring pattern of overexpression of defense related genes in *A. valida* and its hybrid ValSten1, overall comparison of functional annotation in defense-related DEGs between cultivated and wild peanut (AA, BB, and AABB) showed that genes in many categories were underexpressed in wild species than in the case of cultivated peanut (**Table 1**). The host phenotype alteration in the wild species and their hybrid in comparison with the tetraploid cultivars following TSWV infection was not as severe. This could have resulted in less physiological perturbances in the wild diploid species and their hybrid than in the cultivated tetraploids.

In addition to differential expression of defense related genes, other genes such as phytohormones and photosynthesis related genes also were differentially expressed. Altogether, more than half-a-dozen phytohormones were downregulated in *A. stenosperma* and the hybrid ValSten1. Phytohormones were slightly overexpressed in the case of *A. valida*. Phytohormones can induce systemic resistance and inhibit infection of viruses such as TSWV (Zhao et al., 2020). Similarly, the increased flavonoid content facilitated by the overexpression of *SlCHS3* played a significant role in TSWV resistance in tomato plants (Lv et al., 2022). In another study, resistance against the thrips-borne virus in pepper was associated with auxin-related pathway (Zhao et al., 2022). Results in this study showed that genes related to abscisic acid (ABA) and auxin were underexpressed in wild peanut species. Likewise, the DEGs associated with auxin were underexpressed following TSWV infection in susceptible and resistant tomato lines, while DEGs related to ethylene were overexpressed (Lv et al., 2023). In contrast, the miRNA associated with auxin pathways were overexpressed in pepper plants following TSWV infection (Tao et al., 2022). Although ABA plays a role against bacteria and fungi (Alazem and Lin, 2017), virus infection did not result in overexpression of ABA in some incompatible interactions (Kovač et al., 2009; Baetz and Martinoia, 2014). For example, infection by potato virus Y (PVY) of the resistant potato cultivar did not induce ABA (Kazan and Manners, 2009). PVY, like TSWV, is non-tissue specific. Phytohormone gene expression results in this study are in contrast with the tetraploid cultivars examined in another study, wherein phytohormone related genes were overexpressed (Catto et al., 2021). The overexpression was more prominent in the TSWV-resistant cultivar, Tifguard, than in the susceptible cultivar (Catto et al., 2021).

Chloroplast and photosynthesis related genes also were underexpressed overall in both diploids and their hybrid, with the reduced expression being more prominent in *A. stenosperma* followed by the hybrid ValSten1 and *A. valida*. The results were congruent with the other study, in which photosynthesis related genes were underexpressed in both TSWV resistant and susceptible genotypes, with the underexpression being substantial in the case of the TSWV-susceptible cultivar, SunOleic 97R (Catto et al., 2021). Similarly, in the current study, the downregulation of photosynthesis related genes was less substantial in the case of the *A. valida* followed by the hybrid and *A. stenosperma*. These results reiterate that the *A. valida*, and by extension the BB genome, could be more tolerant to thrips-mediated TSWV inoculation.

TSWV resistance in wild diploid species and their hybrids could play a pivotal role in broadening the resistance base against TSWV and possibly other pathogens and pests. The wild diploid species and the hybrid transcriptomes developed in this study provide significant insights into virus-host interactions. Even though, the roles of the differentially expressed genes remain to be functionally validated, DEG analyses provide an overview of the mechanistic underpinning for the observed resistance/tolerance against TSWV. The differential gene expression analyses indicated that defense related genes were consistently overexpressed in the diploid species with the BB genome as opposed to the species with the AA genome. If the pattern remains consistent, then it would be beneficial to focus on wild species such as *A. valida* for enhancing TSWV resistance in cultivated peanut. Further exploration into other molecular factors, such as differential methylation and microRNA expression, in relation to virus resistance in peanut might also be critical (Bertioli et al., 2016; Arora et al., 2022; Tao et al., 2022; Huang et al., 2023).

## Data availability statement

The data for this article can be found in the NCBI GenBank repository at https://www.ncbi.nlm.nih.gov/ under the BioProject PRJNA834809. Raw sequence data for the BioSamples: SAMN28103668-SAMN28103695 are deposited in the SRA accessions: SRR19119579-SRR19119606. The transcriptome shotgun assembly (TSA) submission accessions for *A. valida, A. stenosperma*, and ValSten1 are GJYP00000000, GJYQ00000000, and GJYX00000000 respectively.

## Author contributions

RS: Conceptualization, Funding acquisition, Project administration, Resources, Supervision, Validation, Visualization, Writing – review & editing. YC: Conceptualization, Methodology, Formal Analysis, Software, Writing – review & editing. MC: Data curation, Formal Analysis, Methodology, Software, Writing – review & editing. SP: Data curation, Formal Analysis, Methodology, Software, Writing – review & editing. SL-B: Conceptualization, Funding acquisition, Project administration, Resources, Supervision, Visualization, Writing – review & editing. MA: Project administration, Resources, Supervision, Writing – review & editing. BH: Data curation, Formal Analysis, Writing – review & editing. SB: Supervision, Writing – review & editing. AC: Writing – review & editing.

## Supplementary material

Supplementary material data files 3 and 4 are available via Figshare: 10.6084/m9.figshare.23811546.

## References

[B1] Agarwal G. Clevenger J. Kale S. M. Wang H. Pandey M. K. Choudhary D.. (2019). A recombination bin-map identified a major QTL for resistance to Tomato Spotted Wilt Virus in peanut (Arachis hypogaea). Sci. Rep. 9, 1–13. doi: 10.1038/s41598-019-54747-1 31796847 PMC6890646

[B2] Alazem M. Lin N. S. (20171760). Antiviral roles of abscisic acid in plants. Front. Plant Sci. 8. doi: 10.3389/fpls.2017.01760 PMC5641568 29075279

[B3] Andrews S. FastQC A. (2010). A quality control tool for high throughput sequence data. Available at: http://www.bioinformatics.bbsrc.ac.uk/projects/fastqc/.

[B4] Arora H. Singh R. K. Sharma S. Sharma N. Panchal A. Das T.. (2022). DNA methylation dynamics in response to abiotic and pathogen stress in plants. Plant Cell Rep. 41, 1931–1944. doi: 10.1007/s00299-022-02901-x 35833989

[B5] Baetz U. Martinoia E. (2014). Root exudates: The hidden part of plant defense. Trends Plant Sci. 19, 90–98. doi: 10.1016/j.tplants.2013.11.006 24332225

[B6] Balint-Kurti P. (2019). The plant hypersensitive response: concepts, control and consequences. Mol. Plant Pathol. 20, 1163–1178. doi: 10.1111/mpp.12821 31305008 PMC6640183

[B7] Bertioli D. J. Cannon S. B. Froenicke L. Huang G. Farmer A. D. Cannon E. K. S.. (2016). The genome sequences of Arachis duranensis and Arachis ipaensis, the diploid ancestors of cultivated peanut. Nat. Genet. 484 48, 438–446. doi: 10.1038/ng.3517 26901068

[B8] Bolger A. M. Lohse M. Usadel B. (2014). Trimmomatic: A flexible trimmer for Illumina sequence data. Bioinformatics 30, 2114–2120. doi: 10.1093/bioinformatics/btu170 24695404 PMC4103590

[B9] Boukar O. Fatokun C. A. Huynh B. L. Roberts P. A. Close T. J. (2016). Genomic tools in cowpea breeding programs: Status and perspectives. Front. Plant Sci. 7. doi: 10.3389/fpls.2016.00757 PMC4891349 27375632

[B10] Bushnell B. (2014). BBMap: A fast, accurate, splice-aware aligner. Available at: https://www.osti.gov/biblio/1241166 (Accessed September 26, 2023).

[B11] Camacho C. Coulouris G. Avagyan V. Ma N. Papadopoulos J. Bealer K.. (2009). BLAST+: architecture and applications. BMC Bioinf. 10, 1–9. doi: 10.1186/1471-2105-10-421 PMC2803857 20003500

[B12] Caplan J. L. Mamillapalli P. Burch-Smith T. M. Czymmek K. Dinesh-Kumar S. P. (2008). Chloroplastic protein NRIP1 mediates innate immune receptor recognition of a viral effector. Cell 132, 449–462. doi: 10.1016/j.cell.2007.12.031 18267075 PMC2267721

[B13] Catoni M. Miozzi L. Fiorilli V. Lanfranco L. Accotto G. P. (2009). Comparative analysis of expression profiles in shoots and roots of tomato systemically infected by tomato spotted wilt virus reveals organ-specific transcriptional responses. Mol. Plant-Microbe Interact. 22, 1504–1513. doi: 10.1094/MPMI-22-12-1504 19888816

[B14] Catto M. A. Shrestha A. Abney M. R. Champagne D. E. Culbreath A. K. Leal-Bertioli S. C. M.. (2021). Defense-related gene expression following an orthotospovirus infection is influenced by host resistance in arachis hypogaea. Viruses 13, 1303. doi: 10.3390/v13071303 34372510 PMC8310252

[B15] Chen Y.-J. Pandey S. Catto M. Leal-Bertioli S. Abney M. R. Bag S.. (2023). Evaluation of wild peanut species and their allotetraploids for resistance against thrips and thrips-transmitted tomato spotted wilt orthotospovirus (TSWV). Pathogens 12, 1102. doi: 10.3390/pathogens12091102 37764910 PMC10536083

[B16] Chu Y. Stalker H. T. Marasigan K. Levinson C. M. Gao D. Bertioli D. J.. (2021). Registration of three peanut allotetraploid interspecific hybrids resistant to late leaf spot disease and tomato spotted wilt. J. Plant Regist. 15, 562–572. doi: 10.1002/plr2.20146

[B17] Clevenger J. Chu Y. Chavarro C. Botton S. Culbreath A. Isleib T. G.. (2018). Mapping late leaf spot resistance in peanut (Arachis hypogaea) using QTL-seq reveals markers for marker-assisted selection. Front. Plant Sci. 9. doi: 10.3389/fpls.2018.00083 PMC5807350 29459876

[B18] Conesa A. Götz S. García-Gómez J. M. Terol J. Talón M. Robles M. (2005). Blast2GO: A universal tool for annotation, visualization and analysis in functional genomics research. Bioinformatics 21, 3674–3676. doi: 10.1093/bioinformatics/bti610 16081474

[B19] Core Team R. (2021). R: A language and environment for statistical computing. R foundation for statistical computing. R foundation for statistical computing (Vienna, Austria: R Found. Stat. Comput). Available at: https://www.r-project.org/.

[B20] Culbreath A. K. Srinivasan R. (2011). Epidemiology of spotted wilt disease of peanut caused by Tomato spotted wilt virus in the southeastern U.S. Virus Res. 159, 101–109. doi: 10.1016/j.virusres.2011.04.014 21620508

[B21] Culbreath A. K. Todd J. W. Brown S. L. (2003). Epidemiology and management of tomato spotted wilt in peanut. Annu. Rev. Phytopathol. 41, 53–75. doi: 10.1146/annurev.phyto.41.052002.095522 12704217

[B22] de Oliveira A. S. Boiteux L. S. Kormelink R. Resende R. O. (2018). The Sw-5 gene cluster: Tomato breeding and research toward orthotospovirus disease control. Front. Plant Sci. 9. doi: 10.3389/fpls.2018.01055 PMC6060272 30073012

[B23] Dinesh-Kumar S. P. Baker B. J. (2000). Alternatively spliced N resistance gene transcripts: Their possible role in tobacco mosaic virus resistance. Proc. Natl. Acad. Sci. U. S. A. 97, 1908–1913. doi: 10.1073/pnas.020367497 10660679 PMC26535

[B24] Dinesh-Kumar S. P. Tham W. H. Baker B. J. (2000). Structure-function analysis of the tobacco mosaic virus resistance gene N. Proc. Natl. Acad. Sci. U. S. A. 97, 14789–14794. doi: 10.1073/pnas.97.26.14789 11121079 PMC18997

[B25] Ewels P. Magnusson M. Lundin S. Käller M. (2016). MultiQC: Summarize analysis results for multiple tools and samples in a single report. Bioinformatics 32, 3047–3048. doi: 10.1093/bioinformatics/btw354 27312411 PMC5039924

[B26] Fliegmann J. Mithöfer A. Wanner G. Ebel J. (2004). An ancient enzyme domain hidden in the putative β-glucan elicitor receptor of soybean may play an active part in the perception of pathogen-associated molecular patterns during broad host resistance. J. Biol. Chem. 279, 1132–1140. doi: 10.1074/jbc.M308552200 14578352

[B27] Fraser C. M. Rider L. W. Chapple C. (2005). An expression and bioinformatics analysis of the Arabidopsis serine carboxypeptidase-like gene family. Plant Physiol. 138, 1136–1148. doi: 10.1104/pp.104.057950 15908604 PMC1150427

[B28] Fu L. Niu B. Zhu Z. Wu S. Li W. (2012). CD-HIT: Accelerated for clustering the next-generation sequencing data. Bioinformatics 28, 3150–3152. doi: 10.1093/bioinformatics/bts565 23060610 PMC3516142

[B29] Gao D. Araujo A. C. G. Nascimento E. F. M. B. Chavarro M. C. Xia H. Jackson S. A.. (2021). ValSten: a new wild species derived allotetraploid for increasing genetic diversity of the peanut crop (Arachis hypogaea L.). Genet. Resour. Crop Evol. 68, 1471–1485. doi: 10.1007/s10722-020-01076-2

[B30] Glöckner F. O. Yilmaz P. Quast C. Gerken J. Beccati A. Ciuprina A.. (2017). 25 years of serving the community with ribosomal RNA gene reference databases and tools. J. Biotechnol. 261, 169–176. doi: 10.1016/J.JBIOTEC.2017.06.1198 28648396

[B31] Götz S. García-Gómez J. M. Terol J. Williams T. D. Nagaraj S. H. Nueda M. J.. (2008). High-throughput functional annotation and data mining with the Blast2GO suite. Nucleic Acids Res. 36, 3420–3435. doi: 10.1093/nar/gkn176 18445632 PMC2425479

[B32] Grabherr M. G. Haas B. J. Yassour M. Levin J. Z. Thompson D. A. Amit I.. (2011). Full-length transcriptome assembly from RNA-Seq data without a reference genome. Nat. Biotechnol. 297 29, 644–652. doi: 10.1038/nbt.1883 PMC3571712 21572440

[B33] Hilbers M. P. Rossi A. Finazzi-agrò A. Veldink G. A. Vliegenthart J. F. G.. (1994). The primary structure of a lipoxygenase from the shoots of etiolated lentil seedlings derived from its cDNA. Biochim. Biophys. Acta. 1211, 239–242. doi: 10.1016/0005-2760(94)90275-5 8117753

[B34] Hoffmann K. Qiu W. P. Moyer J. W. (2001). Overcoming host- and pathogen-mediated resistance in tomato and tobacco maps to the M RNA of Tomato spotted wilt virus. Mol. Plant-Microbe Interact. 14, 242–249. doi: 10.1094/MPMI.2001.14.2.242 11204788

[B35] Hong C. E. Ha Y. I. Choi H. Moon J. Y. Lee J. Shin A. Y.. (2017). Silencing of an α-dioxygenase gene, Ca-DOX, retards growth and suppresses basal disease resistance responses in Capsicum annum. Plant Mol. Biol. 93, 497–509. doi: 10.1007/s11103-016-0575-3 28004240

[B36] Huang R. Li H. Gao C. Yu W. Zhang S. (2023). Advances in omics research on peanut response to biotic stresses. Front. Plant Sci. 14. doi: 10.3389/fpls.2023.1101994 PMC10239885 37284721

[B37] Husted L. (1930). Cytological studies on th Peanut, Arachis. II. Chromosom number, morphology and behaviour, and their application to the problem of the origin of the cultivated forms. Cytologia (Tokyo). 7, 396–423.

[B38] Jones P. Binns D. Chang H. Y. Fraser M. Li W. McAnulla C.. (2014). InterProScan 5: Genome-scale protein function classification. Bioinformatics 30, 1236–1240. doi: 10.1093/bioinformatics/btu031 24451626 PMC3998142

[B39] Kanehisa M. Goto S. (2000). KEGG: kyoto encyclopedia of genes and genomes. Nucleic Acids Res. 28, 27–30. doi: 10.1093/nar/28.1.27 10592173 PMC102409

[B40] Kanehisa M. Sato Y. Kawashima M. Furumichi M. Tanabe M. (2016). KEGG as a reference resource for gene and protein annotation. Nucleic Acids Res. 44, D457–D462. doi: 10.1093/nar/gkv1070 26476454 PMC4702792

[B41] Kazan K. Manners J. M. (2009). Linking development to defense: auxin in plant-pathogen interactions. Trends Plant Sci. 14, 373–382. doi: 10.1016/j.tplants.2009.04.005 19559643

[B42] Kopylova E. Noé L. Touzet H. (2012). SortMeRNA: Fast and accurate filtering of ribosomal RNAs in metatranscriptomic data. Bioinformatics 28, 3211–3217. doi: 10.1093/bioinformatics/bts611 23071270

[B43] Kovač M. Müller A. Milovanovič Jarh D. Milavec M. Düchting P. Ravnikar M. (2009). Multiple hormone analysis indicates involvement of jasmonate signalling in the early defence of potato to potato virus YNTN. Biol. Plant 53, 195–199. doi: 10.1007/s10535-009-0034-y

[B44] Lahre K. Shekasteband R. Meadows I. Whitfield A. E. Rotenberg D. (2023). First report of resistance-breaking variants of tomato spotted wilt virus (TSWV) infecting tomatoes with the sw-5 resistance gene in north carolina. Plant Dis. 107, 2271. doi: 10.1094/PDIS-11-22-2637-PDN

[B45] Lai P. C. (2015). Evaluation of cultural tactics, insecticides, and peanut genotypes for thrips and spotted wilt disease management in peanut. MS Thesis, University of Georgia, USA.

[B46] Lai P. C. Abney M. R. Bag S. Culbreath A. K. Srinivasan R. (2021a). Impact of host resistance to tomato spotted wilt orthotospovirus in peanut cultivars on virus population genetics and thrips fitness. Pathogens 10, 1418. doi: 10.3390/pathogens10111418 34832574 PMC8625697

[B47] Lai P. C. Abney M. R. Chen Y. J. Bag S. Srinivasan R. (2021b). Discrepancies in serology-based and nucleic acid-based detection and quantitation of tomato spotted wilt orthotospovirus in leaf and root tissues from symptomatic and asymptomatic peanut plants. Pathogens 10, 1476. doi: 10.3390/pathogens10111476 34832630 PMC8624541

[B48] Langmead B. Salzberg S. L. (2012). Fast gapped-read alignment with Bowtie 2. Nat. Methods 94 9, 357–359. doi: 10.1038/nmeth.1923 PMC3322381 22388286

[B49] Langmead B. Trapnell C. Pop M. Salzberg S. L. (2009). Ultrafast and memory-efficient alignment of short DNA sequences to the human genome. Genome Biol. 10, 1–10. doi: 10.1186/gb-2009-10-3-r25 PMC2690996 19261174

[B50] Langmead B. Wilks C. Antonescu V. Charles R. (2019). Scaling read aligners to hundreds of threads on general-purpose processors. Bioinformatics 35, 421–432. doi: 10.1093/bioinformatics/bty648 30020410 PMC6361242

[B51] Leal-Bertioli S. C. M. Cavalcante U. Gouvea E. G. Ballén-Taborda C. Shirasawa K. Guimarães P. M.. (2015). Identification of QTLs for rust resistance in the peanut wild species Arachis magna and the development of KASP markers for marker-assisted selection. G3 Genes Genomes Genet. 5, 1403–1413. doi: 10.1534/g3.115.018796 PMC4502374 25943521

[B52] Lee K. P. Li M. Li M. Liu K. Medina-Puch L. Qi S.. (2023). Hierarchical regulatory module GENOMES UNCOUPLED1-GOLDEN2-LIKE1/2-WRKY18/40 modulates salicylic acid signaling. Plant Physiol. 192, 3120–3133. doi: 10.1093/plphys/kiad251 37096689

[B53] Levinson C. M. (2021). Morphological, reproductive, genetic, and disease and insect resistance characterization in nascent allotetraploids cross-compatible to cultivated peanut (Arachis hypogaea L.). PhD Thesis, University of Georgia, USA.

[B54] Li B. Dewey C. N. (2011). RSEM: Accurate transcript quantification from RNA-Seq data with or without a reference genome. BMC Bioinformatics 12, 1–16. doi: 10.1186/1471-2105-12-323 21816040 PMC3163565

[B55] Love M. I. Huber W. Anders S. (2014). Moderated estimation of fold change and dispersion for RNA-seq data with DESeq2. Genome Biol. 15, 1–21. doi: 10.1186/s13059-014-0550-8 PMC4302049 25516281

[B56] Lv J. Deng M. Jiang S. Zhu H. Li Z. Wang Z.. (2022). Mapping and functional characterization of the tomato spotted wilt virus resistance gene SlCHS3 in Solanum lycopersicum. Mol. Breed. 42, 1–11. doi: 10.1007/s11032-022-01325-5 37313421 PMC10248591

[B57] Lv J. Deng M. Li Z. Zhu H. Wang Z. Yue Y.. (2023). Integrative analysis of the transcriptome and metabolome reveals the response mechanism to tomato spotted wilt virus. Hortic. Plant J 9 (5), 958–970. doi: 10.1016/j.hpj.2022.12.008

[B58] Lyerly J. H. Stalker H. T. Moyer J. W. Hoffman K. (2002). Evaluation of arachis species for resistance to tomato spotted wilt virus. Peanut Sci. 29, 79–84. doi: 10.3146/pnut.29.2.0001

[B59] Manni M. Berkeley M. R. Seppey M. Zdobnov E. M. (2021). BUSCO: assessing genomic data quality and beyond. Curr. Protoc. 1, e323. doi: 10.1002/cpz1.323 34936221

[B60] Marasigan K. Toews M. Kemerait R. Abney M. R. Culbreath A. Srinivasan R. (2015). Evaluation of alternatives to carbamate and organophosphate insecticides against thrips and tomato spotted wilt virus in peanut production. J. Econ. Entomol. 109, 544–557. doi: 10.1093/jee/tov336 26637534

[B61] Mi H. Muruganujan A. Ebert D. Huang X. Thomas P. D. (2019). PANTHER version 14: More genomes, a new PANTHER GO-slim and improvements in enrichment analysis tools. Nucleic Acids Res. 47, D419–D426. doi: 10.1093/nar/gky1038 30407594 PMC6323939

[B62] Milla S. R. Isleib T. G. Stalker H. T. (2005). Taxonomic relationships among Arachis sect. Arachis species as revealed by AFLP markers. Genome 48, 1–11. doi: 10.1139/G04-089 15729391

[B63] Mishra A. Behura A. Mawatwal S. Kumar A. Naik L. Mohanty S. S.. (2019). Structure-function and application of plant lectins in disease biology and immunity. Food Chem. Toxicol. 134, 110827. doi: 10.1016/J.FCT.2019.110827 31542433 PMC7115788

[B64] Moretzsohn M. C. Gouvea E. G. Inglis P. W. Leal-Bertioli S. C. M. Valls J. F. M. Bertioli D. J. (2013). A study of the relationships of cultivated peanut (Arachis hypogaea) and its most closely related wild species using intron sequences and microsatellite markers. Ann. Bot. 111, 113–126. doi: 10.1093/AOB/MCS237 23131301 PMC3523650

[B65] Moury B. Palloix A. Gebre Selassie K. Marchoux G. (1997). Hypersensitive resistance to tomato spotted wilt virus in three capsicum chinense accessions is controlled by a single gene and is overcome by virulent strains. Euphytica 94, 45–52. doi: 10.1023/A:1002997522379

[B66] Noman A. Liu Z. Aqeel M. Zainab M. Khan M. I. Hussain A.. (2017). Basic leucine zipper domain transcription factors: the vanguards in plant immunity. Biotechnol. Lett. 3912 39, 1779–1791. doi: 10.1007/S10529-017-2431-1 28879532

[B67] Pandey M. K. Monyo E. Ozias-Akins P. Liang X. Guimarães P. Nigam S. N.. (2012). Advances in Arachis genomics for peanut improvement. Biotechnol. Adv. 30, 639–651. doi: 10.1016/J.BIOTECHADV.2011.11.001 22094114

[B68] Pandey S. P. Somssich I. E. (2009). The role of WRKY transcription factors in plant immunity. Plant Physiol. 150, 1648–1655. doi: 10.1104/PP.109.138990 19420325 PMC2719123

[B69] Pandey M. K. Wang H. Khera P. Vishwakarma M. K. Kale S. M. Culbreath A. K.. (2017). Genetic dissection of novel QTLs for resistance to leaf spots and tomato spotted wilt virus in peanut (Arachis hypogaea L.). Front. Plant Sci. 8. doi: 10.3389/FPLS.2017.00025/BIBTEX PMC5281592 28197153

[B70] Rodríguez-Negrete E. A. Guevara-Rivera E. A. Arce-Leal Á.P. Leyva-López N. E. Méndez-Lozano J. (2023). A novel tomato spotted wilt virus isolate encoding a noncanonical NSm C118F substitution associated with Sw-5 tomato gene resistance breaking. Mol. Plant Pathol. 24, 1300–1311. doi: 10.1111/MPP.13371 37403515 PMC10502823

[B71] Rossi M. Goggin F. L. Milligan S. B. Kaloshian I. Ullman D. E. Williamson V. M. (1998). The nematode resistance gene Mi of tomato confers resistance against the potato aphid. Proc. Natl. Acad. Sci. U. S. A. 95, 9750–9754. doi: 10.1073/pnas.95.17.9750 9707547 PMC21408

[B72] Rotenberg D. Kumar N. K. K. Ullman D. E. Montero-Astúa M. Willis D. K. German T. L.. (2009). Variation in Tomato spotted wilt virus titer in Frankliniella occidentalis and its association with frequency of transmission. Phytopathology 99, 404–410. doi: 10.1094/PHYTO-99-4-0404 19271982

[B73] Roux F. Bergelson J. (2016). The genetics underlying natural variation in the biotic interactions of arabidopsis thaliana: the challenges of linking evolutionary genetics and community ecology. Curr. Top. Dev. Biol. 119, 111–156. doi: 10.1016/BS.CTDB.2016.03.001 27282025

[B74] Santana S. H. Valls J. F. M. (2015). Arachis veigae. Bonplandia 24, 139–150. doi: 10.30972/bon.242238

[B75] Seijo G. J. Atahuachi M. Simpson C. E. Krapovickas A. (2021). Arachis inflata: A New B Genome species of Arachis (Fabaceae). Bonplandia 30, 169–174. doi: 10.30972/BON.3024942

[B76] Seppey M. Manni M. Zdobnov E. M. (2019). BUSCO: Assessing genome assembly and annotation completeness. Methods Mol. Biol. 1962, 227–245. doi: 10.1007/978-1-4939-9173-0_14 31020564

[B77] Shrestha A. Champagne D. E. Culbreath A. K. Rotenberg D. Whitfield A. E. Srinivasan R. (2017). Transcriptome changes associated with Tomato spotted wilt virus infection in various life stages of its thrips vector, Frankliniella fusca (Hinds). J. Gen. Virol. 98, 2156–2170. doi: 10.1099/jgv.0.000874 28741996

[B78] Shrestha A. Srinivasan R. Riley D. G. Culbreath A. K. (2012). Direct and indirect effects of a thrips-transmitted Tospovirus on the preference and fitness of its vector, Frankliniella fusca. Entomol. Exp. Appl. 145, 260–271. doi: 10.1111/EEA.12011

[B79] Shrestha A. Srinivasan R. Sundaraj S. Culbreath A. K. Riley D. G. (2013). Second generation peanut genotypes resistant to thrips-transmitted tomato spotted wilt virus exhibit tolerance rather than true resistance and differentially affect thrips fitness. J. Econ. Entomol. 106, 587–596. doi: 10.1603/EC12430 23786043

[B80] Shrestha A. Sundaraj S. Culbreath A. K. Riley D. G. Abney M. R. Srinivasan R. (2015). Effects of thrips density, mode of inoculation, and plant age on tomato spotted wilt virus transmission in peanut plants. Environ. Entomol. 44, 136–143. doi: 10.1093/EE/NVU013 26308816

[B81] Simão F. A. Waterhouse R. M. Ioannidis P. Kriventseva E. V. Zdobnov E. M. (2015). BUSCO: assessing genome assembly and annotation completeness with single-copy orthologs. Bioinformatics 31, 3210–3212. doi: 10.1093/BIOINFORMATICS/BTV351 26059717

[B82] Simpson C. E. (1991). Pathways for Introgression of Pest Resistance into Arachis hypogaea L. Peanut Sci. 18, 22–26. doi: 10.3146/I0095-3679-18-1-8

[B83] Slater G. S. C. Birney E. (2005). Automated generation of heuristics for biological sequence comparison. BMC Bioinf. 6, 1–11. doi: 10.1186/1471-2105-6-31 PMC553969 15713233

[B84] Song J. Bradeen J. M. Naess S. K. Raasch J. A. Wielgus S. M. Haberlach G. T.. (2003). Gene RB cloned from Solanum bulbocastanum confers broad spectrum resistance to potato late blight. Proc. Natl. Acad. Sci. U. S. A. 100, 9128–9133. doi: 10.1073/PNAS.1533501100 12872003 PMC170883

[B85] Srinivasan R. Abney M. R. Culbreath A. K. Kemerait R. C. Tubbs R. S. Monfort W. S.. (2017). Three decades of managing Tomato spotted wilt virus in peanut in southeastern United States. Virus Res. 241, 203–212. doi: 10.1016/J.VIRUSRES.2017.05.016 28549856

[B86] Srinivasan R. Abney M. R. Lai P. C. Culbreath A. K. Tallury S. Leal-Bertioli S. C. M. (2018). Resistance to thrips in peanut and implications for management of thrips and thrips-transmitted orthotospoviruses in peanut. Front. Plant Sci. 871. doi: 10.3389/FPLS.2018.01604/BIBTEX PMC6232880 30459792

[B87] Stalker H. T. (2017). Utilizing wild species for peanut improvement. Crop Sci. 57, 1102–1120. doi: 10.2135/CROPSCI2016.09.0824

[B88] Stalker H. T. Campbell W. V. (1983). Resistance of wild species of peanut to an insect complex1. Peanut Sci. 10, 30–33. doi: 10.3146/i0095-3679-10-1-9

[B89] Stevens M. R. Scott S. J. Gergerich R. C. (1991). Inheritance of a gene for resistance to tomato spotted wilt virus (TSWV) from Lycopersicon Peruvianum Mill. Euphytica 59, 9–17. doi: 10.1007/BF00025356

[B90] Sundaraj S. Srinivasan R. Culbreath A. K. Riley D. G. Pappu H. R. (2014). Host Plant Resistance Against Tomato spotted wilt virus in Peanut (Arachis hypogaea) and Its Impact on Susceptibility to the Virus, Virus Population Genetics, and Vector Feeding Behavior and Survival. Virology 104, 202–210. doi: 10.1094/PHYTO-04-13-0107-R 24025049

[B91] Supek F. Bošnjak M. Škunca N. Šmuc T. (2011). Revigo summarizes and visualizes long lists of gene ontology terms. PloS One 6, e21800. doi: 10.1371/journal.pone.0021800 21789182 PMC3138752

[B92] Takabatake R. Karita E. Seo S. Mitsuhara I. Kuchitsu K. Ohashi Y. (2007). Pathogen-induced calmodulin isoforms in basal resistance against bacterial and fungal pathogens in tobacco. Plant Cell Physiol. 48, 414–423. doi: 10.1093/pcp/pcm011 17251204

[B93] Tao H. Jia Z. Gao X. Gui M. Li Y. Liu Y. (2022). Analysis of the miRNA expression profile involved in the tomato spotted wilt orthotospovirus–pepper interaction. Virus Res. 312, 198710. doi: 10.1016/j.virusres.2022.198710 35183573

[B94] Tseng Y. C. Tillman B. L. Peng Z. Wang J. (2016). Identification of major QTLs underlying tomato spotted wilt virus resistance in peanut cultivar Florida-EPTM “113.”. BMC Genet. 17, 1–14. doi: 10.1186/s12863-016-0435-9 27600750 PMC5012072

[B95] Ullman D. E. (1992). A midgut barrier to tomato spotted wilt virus acquisition by adult western flower thrips. Phytopathology 82, 1333. doi: 10.1094/phyto-82-1333

[B96] Valls J. F. M. Da Costa L. C. Custódio A. R. (2013). A novel trifoliolate species of Arachis (Fabaceae) and further comments on taxonomic section Trierectoides. Bonplandia 22, 91–97. doi: 10.30972/bon.2211257

[B97] Valls J. F. M. Simpson C. E. (2005). New species of arachis (Leguminosae) from Brazil, Paraguay and Bolivia. Bonplandia 14, 35–63. doi: 10.30972/bon.141-21387

[B98] Valls J. F. M. Simpson C. E. (2017). A new species of Arachis (Fabaceae) from Mato Grosso, Brazil, related to Arachis matiensis. Bonplandia 26, 143–149. doi: 10.30972/bon.2622575

[B99] Van Der Vossen E. Sikkema A. Te Lintel Hekkert B. Gros J. Stevens P. Muskens M.. (2003). An ancient R gene from the wild potato species Solanum bulbocastanum confers broad-spectrum resistance to Phytophthora infestans in cultivated potato and tomato. Plant J. 36, 867–882. doi: 10.1046/j.1365-313X.2003.01934.x 14675451

[B100] Whitham S. Dinesh-Kumar S. P. Choi D. Hehl R. Corr C. Baker B. (1994). The product of the tobacco mosaic virus resistance gene N: Similarity to toll and the interleukin-1 receptor. Cell 78, 1101–1115. doi: 10.1016/0092-8674(94)90283-6 7923359

[B101] Wu T. D. Reeder J. Lawrence M. Becker G. Brauer M. J. (2016). GMAP and GSNAP for genomic sequence alignment: Enhancements to speed, accuracy, and functionality. Methods Mol. Biol. 1418, 283–334. doi: 10.1007/978-1-4939-3578-9_15 27008021

[B102] Wu X. Zhang X. Wang H. Fang R. X. Ye J. (2023). Structure–function analyses of coiled-coil immune receptors define a hydrophobic module for improving plant virus resistance. J. Exp. Bot. 74, 1372–1388. doi: 10.1093/jxb/erac477 36472617 PMC10010612

[B103] Xu L. Dong Z. Fang L. Luo Y. Wei Z. Guo H.. (2019). OrthoVenn2: a web server for whole-genome comparison and annotation of orthologous clusters across multiple species. Nucleic Acids Res. 47, W52–W58. doi: 10.1093/NAR/GKZ333 31053848 PMC6602458

[B104] Yilmaz P. Parfrey L. W. Yarza P. Gerken J. Pruesse E. Quast C.. (2014). The SILVA and “all-species Living Tree Project (LTP)” taxonomic frameworks. Nucleic Acids Res. 42, D643–D648. doi: 10.1093/nar/gkt1209 24293649 PMC3965112

[B105] Yu C. K. Y. Springob K. Schmidt J. Nicholson R. L. Chu I. K. Wing K. Y.. (2005). A stilbene synthase gene (SbSTS1) is involved in host and nonhost defense responses in orghum. Plant Physiol. 138, 393–401. doi: 10.1104/PP.105.059337 15821144 PMC1104192

[B106] Zhang C. Xie W. Fu H. Chen Y. Chen H. Cai T.. (2023). Whole genome resequencing identifies candidate genes and allelic diagnostic markers for resistance to Ralstonia solanacearum infection in cultivated peanut (Arachis hypogaea L.). Front. Plant Sci. 13. doi: 10.3389/fpls.2022.1048168 PMC9845939 36684803

[B107] Zhao L. Hu Z. Li S. Zhang L. Yu P. Zhang J.. (2020). Tagitinin A from Tithonia diversifolia provides resistance to tomato spotted wilt orthotospovirus by inducing systemic resistance. Pestic. Biochem. Physiol. 169, 104654. doi: 10.1016/J.PESTBP.2020.104654 32828372

[B108] Zhao Z. Tseng Y. C. Peng Z. Lopez Y. Chen C. Y. Tillman B. L.. (2018). Refining a major QTL controlling spotted wilt disease resistance in cultivated peanut (Arachis hypogaea L.) and evaluating its contribution to the resistance variations in peanut germplasm. BMC Genet. 19. doi: 10.1186/S12863-018-0601-3 PMC5865372 29571286

[B109] Zhao L. Zhang L. Hu Z. Li B. Zheng X. Qiu R.. (2022). Tomato zonate spot virus induced hypersensitive resistance *via* an auxin-related pathway in pepper. Gene 823. doi: 10.1016/j.gene.2022.146320 35218893

