# Supplementary material for: Characterization of gene expression patterns in response to an orthotospovirus infection between two diploid peanut species and their hybrid
Source: Front Plant Sci. 2023 Nov 13;14:1270531. doi: 10.3389/fpls.2023.1270531 (PMC10683084; doi:10.3389/fpls.2023.1270531)
Supplement: Supplementary file 1 [file DataSheet_1.docx]

Supplementary Material

# Characterization of Gene Expression Patterns Associated with TSWV Resistance Between Diploid and Tetraploid Peanut Species

**Yi-Ju Chen^1, †^, Michael A. Catto^1, †^, Sudeep Pandey^1^, Soraya Leal-Bertioli^2^, Mark Abney^3^, Brendan G. Hunt^1^, Sudeep Bag^4^, and Rajagopalbabu Srinivasan^1, *^**

*** Correspondence:** Rajagopalbabu Srinivasan: [babusri@uga.edu](mailto:babusri@uga.edu)

**Table S1. Gene count estimates from the mapped reads of *Arachis stenosperma.* See Additional file 2.**

**Table S2. Gene count estimates from the mapped reads of *Arachis valida.* See Additional file 2.**

**Table S3. Gene count estimates from the mapped reads of ValSten1*.* See Additional file 2.**

**Table S4. Annotation file of the *A. stenosperma de novo* assembly*.* See Additional file 3.**

**Table S5. Annotation file of the *A. valida de novo* assembly*.* See Additional file 3.**

**Table S6. Annotation file of the ValSten1 *de novo* assembly*.* See Additional file 3.**

**Table S7. Primer pairs designed for normalized quantification on differentially expressed genes (DEGs) in response to tomato spotted wilt orthotospovirus (TSWV) infection in *Arachis* genotypes**

| Genotype | Transcript ID | Reverse Sequence (5'->3') |
| --- | --- | --- |
| Valida^2x^ | AravalEVm003201t1 | TATCTTTGGGCGCCACTTGT |
|  |  | TCGGTCCTGCAATGCTTCAT |
| Valida^2x^ | AravalEVm008478t7 | TGGTGTGGTCTGGAATGGTT |
|  |  | TGCACAATGTGGCTCAACAG |
| Valida^2x^ | AravalEVm008254t5 | CCGTGGCTACACACTTACCC |
|  |  | TATGGCCGAAGCAAGCATGA |
| ValSten1^4x^ | AravsEVm001669t2 | TTGCTAGGACATTTGCCGGA |
|  |  | GCCAAAGGCGAACACATCAG |
| ValSten1^4x^ | AravsEVm021416t2 | CTCCAGATGCGACACCTGTT |
|  |  | GCGCCAGTTGCTTAATTCGT |
| ValSten1^4x^ | AravsEVm011036t2 | TGACGGTTAGAACGGCAACA |
|  |  | TTGAGGGCAGCCACTGTAAT |
| Sten^2x^ | ArasteEVm017297t1 | CCACGAGGGTGAGTTGTTGT |
|  |  | GCAATAAACGGGCGAACACC |
| Sten^2x^ | ArasteEVm003747t1 | GGATGCAGGGGCAATTTCGG |
|  |  | ATCTCCGGCAGTGGCTTTCA |
| Sten^2x^ | ArasteEVm003896t4 | CTGGGCGGGTGAGTATGGAG |
|  |  | TTGGAAGGGCAGAAGTCGCA |
| Peanut | (Internal reference) | GACGCTTGGCGAGATCAACA |
|  |  | AACCGGACAACCACCACATG |

**Table S8. Summary of the RNASeq dataset generated from *Arachis stenosperma*, *Arachis valida*, and ValSten1 mock-inoculated and TSWV-infected TSWV plants**

| Sample ID | Number of Input Reads | Number of Uniquely Mapped Reads | Number of Multiply Mapped Reads | Total Percentage Mapped to Assembly |
| --- | --- | --- | --- | --- |
| asten_paired_M3 | 29,368,534 | 9,988,009 | 15,697,451 | 87.5% |
| asten_paired_M6 | 19,140,614 | 6,529,068 | 9,439,229 | 83.4% |
| asten_paired_M7 | 20,035,941 | 6,908,722 | 9,848,170 | 83.6% |
| asten_paired_M8 | 19,636,769 | 6,572,595 | 10,378,289 | 86.3% |
| asten_paired_M9B | 13,343,007 | 4,358,975 | 6,828,647 | 83.8% |
| asten_paired_V11 | 20,645,348 | 6,879,846 | 11,002,955 | 86.6% |
| asten_paired_V3B | 25,261,650 | 9,031,296 | 13,139,371 | 87.8% |
| asten_paired_V4 | 21,704,792 | 7,995,981 | 10,894,302 | 87.0% |
| asten_paired_V5 | 26,158,639 | 9,325,634 | 13,595,665 | 87.6% |
| availida_paired_M11 | 25,039,985 | 8,035,875 | 13,637,503 | 86.6% |
| availida_paired_M3B | 23,990,954 | 8,010,782 | 13,041,759 | 87.8% |
| availida_paired_M4 | 28,464,507 | 8,893,077 | 16,095,196 | 87.8% |
| availida_paired_M9 | 24,543,724 | 7,970,176 | 13,614,611 | 87.9% |
| availida_paired_V2 | 22,322,834 | 8,431,916 | 10,642,835 | 85.4% |
| availida_paired_V3 | 22,577,536 | 7,675,313 | 12,046,347 | 87.4% |
| availida_paired_V4B | 21,781,422 | 7,255,017 | 11,934,059 | 88.1% |
| availida_paired_V5 | 26,397,622 | 9,369,474 | 14,042,895 | 88.7% |
| availida_paired_V6 | 26,567,169 | 9,513,309 | 13,498,361 | 86.6% |
| avs_paired_M1 | 34,029,958 | 9,465,683 | 18,113,337 | 81.0% |
| avs_paired_M12B | 23,513,720 | 6,846,594 | 12,241,433 | 81.2% |
| avs_paired_M2 | 30,896,630 | 8,387,837 | 16,486,540 | 80.5% |
| avs_paired_M7 | 22,442,379 | 6,438,906 | 11,587,985 | 80.3% |
| avs_paired_M9B | 19,626,010 | 5,594,556 | 10,396,012 | 81.5% |
| avs_paired_V11B | 21,492,612 | 5,948,362 | 10,671,202 | 77.3% |
| avs_paired_V2 | 24,191,325 | 7,259,928 | 12,448,410 | 81.5% |
| avs_paired_V3 | 19,856,626 | 5,364,146 | 10,729,067 | 81.0% |
| avs_paired_V6B | 20,691,308 | 6,476,137 | 10,001,928 | 79.6% |
| avs_paired_V7 | 21,536,150 | 6,578,758 | 10,786,593 | 80.6% |

**Table S9. Counts and statistics of significantly enriched GO terms from TSWV-infected compared to non-infected *A. stenosperma.* See Additional file 4.**

**Table S10. Gene list from the significantly enriched GO terms from TSWV-infected compared to non-infected *A. stenosperma.* See Additional file 4.**

**Table S11. Counts and statistics of significantly enriched GO terms from TSWV-infected compared to non-infected *A. valida.* See Additional file 4.**

**Table S12. Gene list from the significantly enriched GO terms from TSWV-infected compared to non-infected *A. valida.* See Additional file 4.**

**Table S13. Counts and statistics of significantly enriched GO terms from TSWV-infected compared to non-infected ValSten1*.* See Additional file 4.**

**Table S14. Gene list from the significantly enriched GO terms from TSWV-infected compared to non-infected ValSten1*.* See Additional file 4.**

**Table S15. Orthologous clusters of DEGs that were commonly shared by at least two genotypes*.* See Additional file 5.**

**Table S16. Single-copy gene clusters of DEGs from all five genotypes*.* See Additional file 5.**

**Table S17. Wild peanut single-copy orthologous gene clusters of DEGs*.* See Additional file 5.**


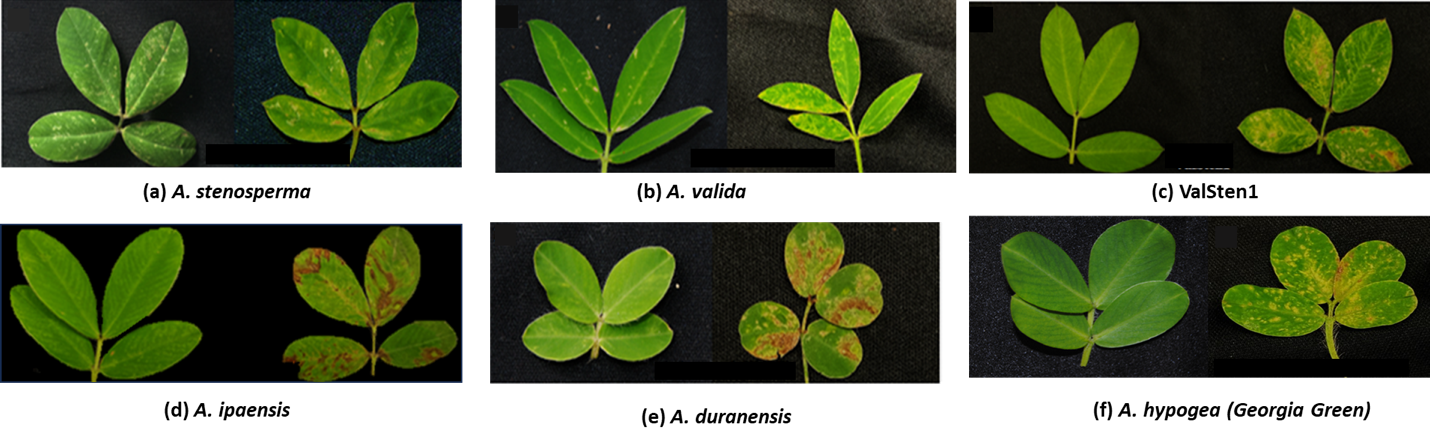


**Figure S1. TSWV-induced symptoms on diploid *Arachis* species and their hybrid**: (**a**) *A. stenosperma* (**b**) *A. valida,* (**c**) ValSten1, (**d**) *A. ipaensis*, (**e**) *A. duranensis*, and (**g**) *A. hypogea* (Georgia Green). Left photograph represents a non-infected leaf, and right photograph represents a TSWV-infected leaf after two weeks of thrips-mediated inoculation including infected and non-infected plants.


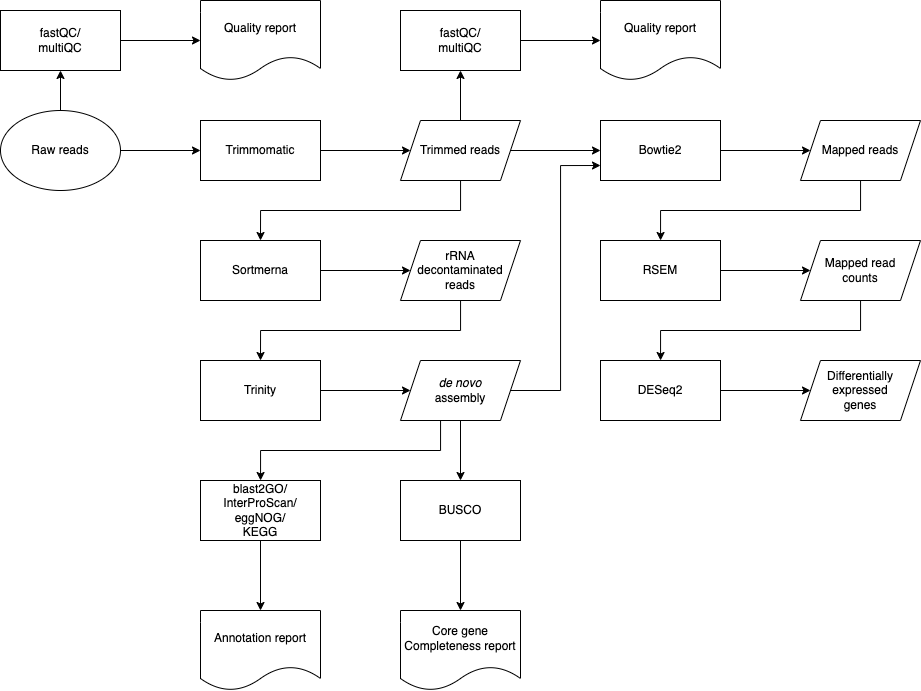


**Figure S2. Pipeline for raw read processing, transcriptome assembly, and mapping and differential expression.** Tools within the pipeline were applied to each of the species of interest: *A. valida*, *A. stenosperma*, and *A. valida* × *A. stenosperma*.


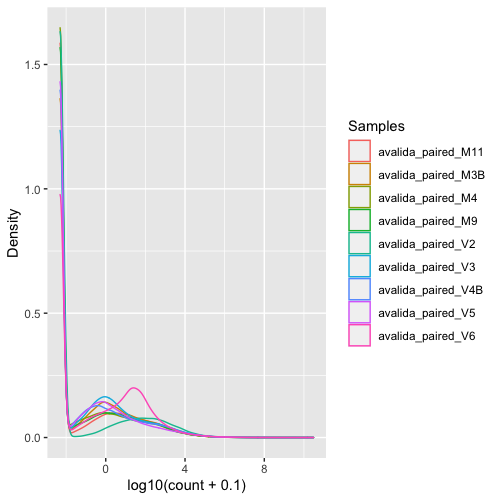
**Figure S3. FPKM distribution from all *A. valida* samples.**


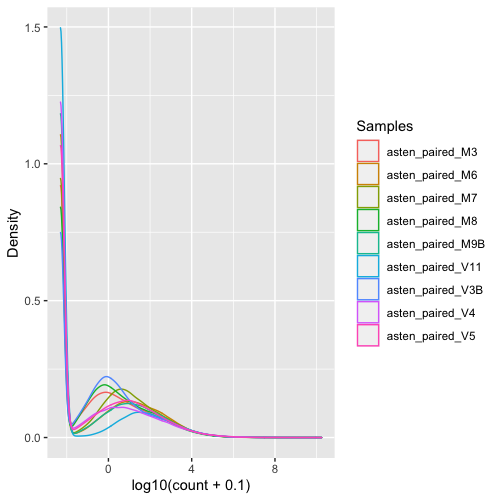
**Figure S4. FPKM distribution from all *A. stenosperma* samples.**


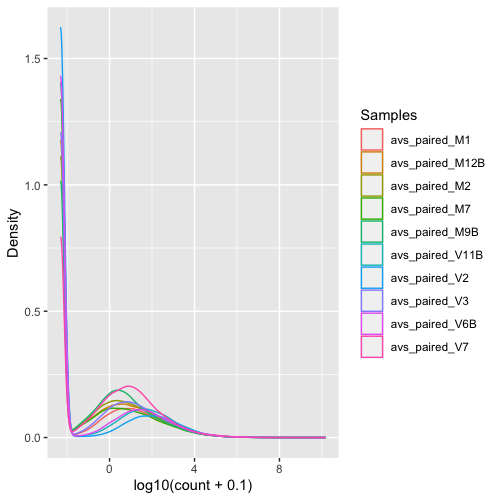
**Figure S5. FPKM distribution from all ValSten1 samples.**


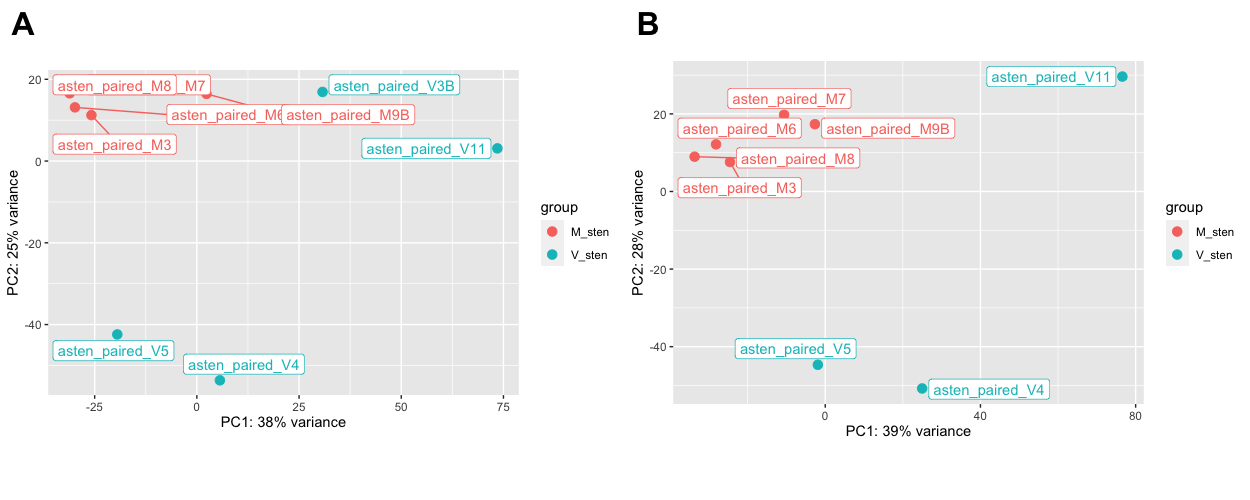
**Figure S6. Principal component analysis of *A. stenosperma* genotypes.** (**A**) *A. stenosperma* V10309 and (**B**) *A. stenosperma* V10309 (“asten_paired_V3B” removed) clustered together according to being either Mock-inoculated (M, in red color) or TSWV-infected (V, in blue color).

**Figure S7. Principal component analysis of *A. stenosperma*, *A. valida*, and ValSten1 with respect to the ValSten1 *de novo* assembly.** PC1 shows separation by genotype, with the hybrid ValSten1 in the middle, where PC2 is primarily separating by infection status: Mock-inoculated (Mock, in red color) or TSWV-infected (Viruliferous, in blue color).

**
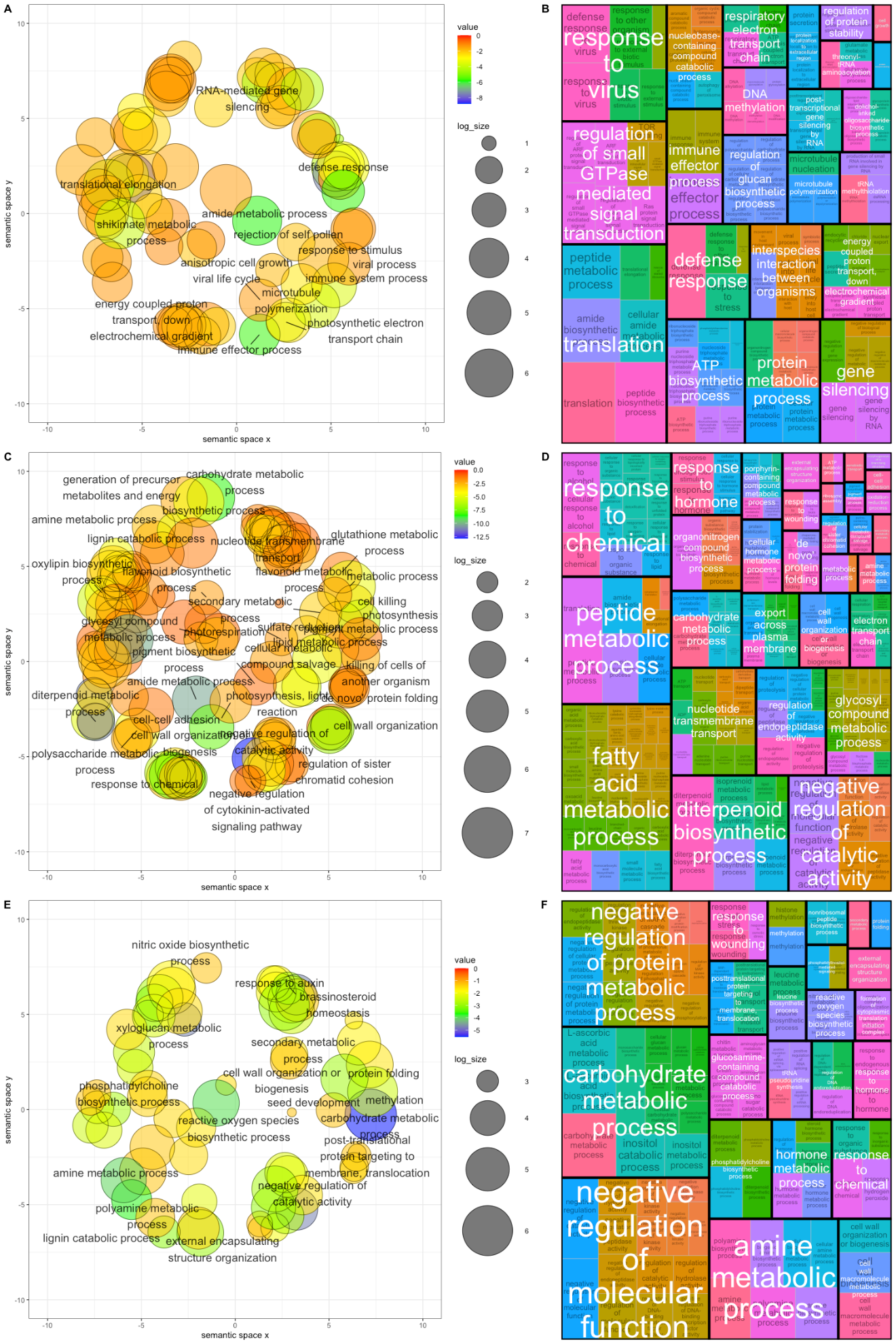
**

**Figure S8. Gene Ontology (GO) terms ratios across genotypes.** (**A**) Ratio of all significant GO terms assigned to differentially expressed genes (DEGs) present in *A. stenosperma.* (**B**) Tree map of all levels of significant GO terms of DEGs compared to the background in *A. stenosperma*. (**C**) Ratio of all significant GO terms assigned to differentially expressed genes (DEGs) present in *A. valida.* (**D**) Tree map of all levels of significant GO terms of DEGs compared to the background in *A. valida*. (**E**) Ratio of all significant GO terms assigned to differentially expressed genes (DEGs) present in ValSten1. (**F**) Tree map of all levels of significant GO terms of DEGs compared to the background in ValSten1.


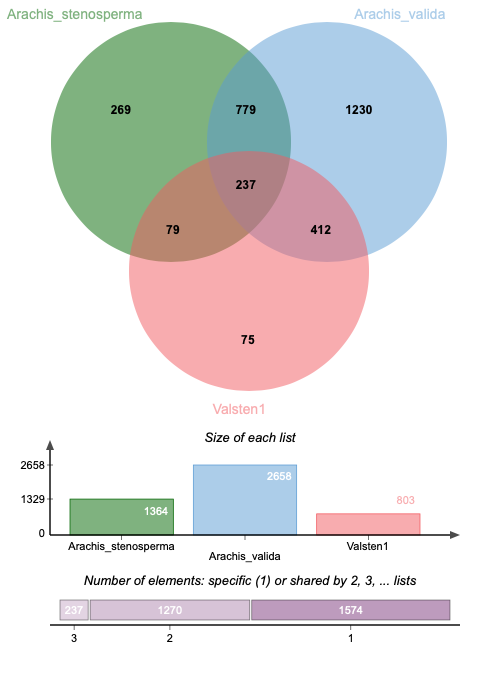


**Figure S9. Overlapping orthologous gene families containing differentially expressed genes across wild peanut (*Arachis spp*.) in response to TSWV infection.** Protein count within gene families (cluster count) across the three wild peanut species *A.* *stenosperma*, *A. valida,* and ValSten1 (*A. valida* × *A.* *stenosperma*).


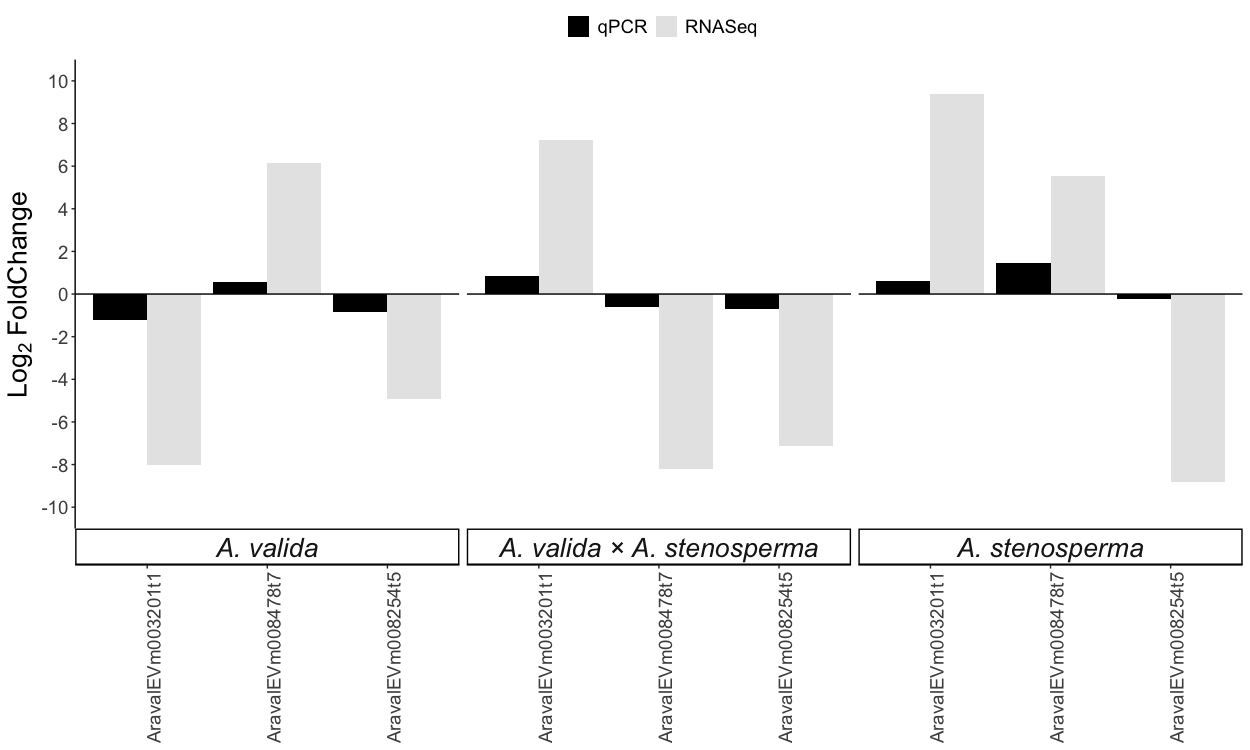


**Figure S10. Quantitative PCR validation of the RNA-Seq analysis using selected differentially expressed Genes (DEGs) in three *Arachis* genotypes in response to TSWV infection.**
